# Recent Advances in the Development of Pyrazole Derivatives as Anticancer Agents

**DOI:** 10.3390/ijms241612724

**Published:** 2023-08-12

**Authors:** Yingqian Zhang, Chenyuan Wu, Nana Zhang, Rui Fan, Yang Ye, Jun Xu

**Affiliations:** 1 School of Pharmacy, Hangzhou Normal University, Hangzhou 311121, China; wu41243107@163.com (C.W.); znn74438@163.com (N.Z.); fr6987321@163.com (R.F.); yeyang0711@163.com (Y.Y.); 2 Collaborative Innovation Center of Traditional Chinese Medicines of Zhejiang Province, Key Laboratory of Elemene Class Anti-Cancer Chinese Medicines, Engineering Laboratory of Development and Application of Traditional Chinese Medicines, Hangzhou Normal University, Hangzhou 311121, China; 3 College of Material, Chemistry and Chemical Engineering, Key Laboratory of Organosilicon Chemistry and Material Technology, Ministry of Education, Hangzhou Normal University, Hangzhou 311121, China

**Keywords:** pyrazole, anticancer, cytotoxicity, apoptosis, inhibitor

## Abstract

Pyrazole derivatives, as a class of heterocyclic compounds, possess unique chemical structures that confer them with a broad spectrum of pharmacological activities. They have been extensively explored for designing potent and selective anticancer agents. In recent years, numerous pyrazole derivatives have been synthesized and evaluated for their anticancer potential against various cancer cell lines. Structure–activity relationship studies have shown that appropriate substitution on different positions of the pyrazole ring can significantly enhance anticancer efficacy and tumor selectivity. It is noteworthy that many pyrazole derivatives have demonstrated multiple mechanisms of anticancer action by interacting with various targets including tubulin, EGFR, CDK, BTK, and DNA. Therefore, this review summarizes the current understanding on the structural features of pyrazole derivatives and their structure-activity relationships with different targets, aiming to facilitate the development of potential pyrazole-based anticancer drugs. We focus on the latest research advances in anticancer activities of pyrazole compounds reported from 2018 to present.

## 1. Introduction

With rising living standards, the incidence of cancer has exhibited an upward trend, emerging as a leading threat to human health [1,2]. While conventional therapies including surgery and radiotherapy are remarkably efficacious against certain malignancies, their curative effects on aggressive tumors remain limited. As a principal modality of multidisciplinary cancer management, chemotherapy is hampered by considerable toxicities of numerous anticancer agents, severely compromising patients’ quality of life [3]. Therefore, the development of anticancer drugs with high selectivity and low toxicity has become an imperative in medicinal chemistry.

Natural products play a pivotal role in anticancer drug research and development. Numerous naturally occurring heterocycles demonstrate unique antitumor activities, attributed to their structural rigidity imparted by distinct spatial conformations that mediate specific binding to cancer targets. Common heterocyclic scaffolds include pyrrole, furan, pyrazole, etc. [4]. Notably, pyrazoles and their derivatives have exhibited tremendous application potential in the discovery of novel anticancer therapeutics, owing to their unique chemical structures and remarkable bioactivities.

Pyrazole is an important five-membered aromatic heterocyclic compound. Its unique chemical structure endows pyrazole with tremendous application value. The pyrazole ring consists of three adjacent carbon atoms and two nitrogen atoms, which impart polarity and reactivity to pyrazole due to the presence of nitrogen atoms. In addition, the pyrazole ring can undergo various substitution reactions. This provides ample opportunities for designing and developing novel anticancer drugs with pyrazole as the core scaffold.

Pyrazole and its derivatives have demonstrated broad pharmacological activities, including anti-inflammatory, analgesic [5], antimicrobial [6], anticonvulsant [7], anticancer [8], antitubercular [9], etc. Several pyrazole-derived drugs have been approved for clinical use, including celecoxib, lonazolac, difenamizole, fezolamine, crizotinib, and pyrazofurin. These drugs exhibit diverse pharmacological activities, and selected examples along with their mechanisms of action are presented in Table 1.

Numerous studies have shown that pyrazole derivatives possess good inhibitory activities against various targets in cancer cells, such as EGFR, VEGFR-2, CDK, BTK, and BRAF V600E, etc., which play an important role in anticancer drugs (details are provided below). In recent years, a number of pyrazole derivatives have been designed, synthesized, and evaluated for their anticancer efficacies in order to develop new anticancer candidates. Substituted pyrazoles, pyrazolines, pyrazolones, and pyrazolopyridines are several important classes of pyrazole derivatives. Among them, pyrazoline is a dihydro derivative of pyrazole, containing two adjacent nitrogen atoms and one endocyclic double bond in the ring. Pyrazolopyrimidine is one of the attractive fused heterocyclic pyrazole compounds. Pyrazolone is formed by attaching a ketone group at the 3-position of the pyrazole ring, retaining the coplanar structure of the pyrazole ring. The aforementioned three classes of compounds possess potent anticancer activity, as summarized in recently published reviews [26,27,28]. In this review, we mostly focused on the research results regarding the anticancer activity of substituted pyrazole derivatives published since 2018, and categorize various pyrazole inhibitors based on their different targets of action.

## 2. Tubulin Polymerization Inhibitors

Microtubules are common hollow tubular fibrous structures in cells composed of tubulin, a small protein subunit [29]. Tubulin plays a crucial role in proliferation, trafficking, signaling, and migration in eukaryotic cells by polymerizing into microtubules. Therefore, it has become an important target for antitumor drug design [30]. Tumor cells proliferate rapidly through cycles of tubulin polymerization and depolymerization of tubulin. Compounds that inhibit tubulin polymerization have thus been developed as cytotoxic drugs and are an integral part of chemotherapy [31]. Colchicine was the first compound that was identified to target microtubules, which binds to tubulin and inhibits microtubule polymerization [32].

**Figure 1:**
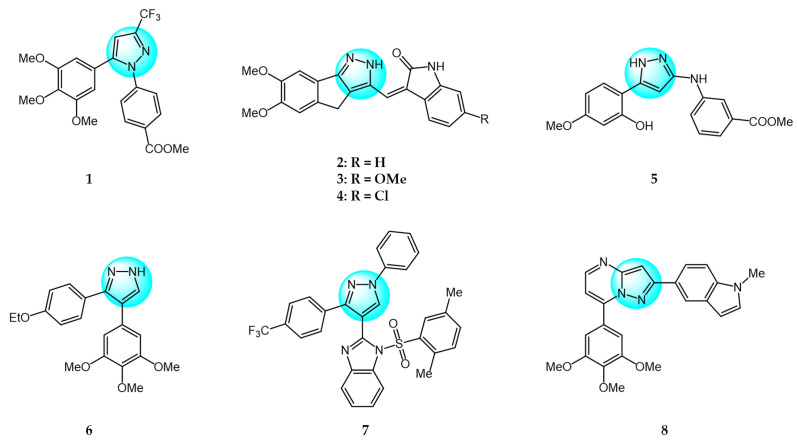

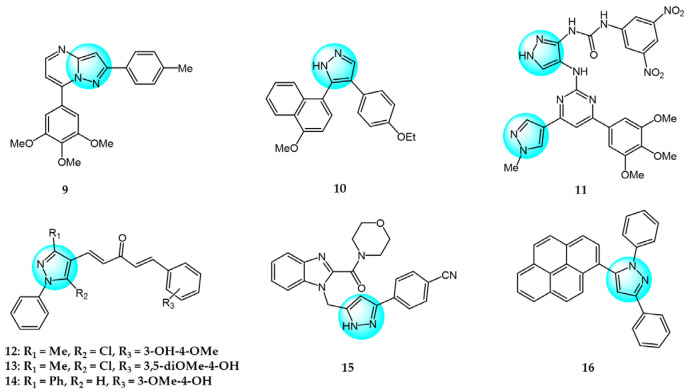
Pyrazole-containing tubulin polymerization inhibitors (compounds **1**–**16**).

Combretastatin A-4 (CA-4) is another well-known antitubulin agent that binds to tubulin and induces microtubule depolymerization. Due to its important pharmacological effects and simple structure, it is a potential lead molecule for designing novel anticancer compounds. Hura et al. synthesized twenty-three hybrid analogs of combretastatin-(trifluoromethyl) pyrazole as potential tubulin polymerization inhibitors by combining the *N*-heterocyclic motif in celecoxib with the key structural features of CA-4 [33]. The antiproliferative activity of the compounds was evaluated in MCF7 breast carcinoma cells. The results showed compound **1** (Figure 1 for structures of compounds **1**–**16**) displayed potent and selective antiproliferative activity across multiple cancer cell lines, including breast (MCF7, IC_50_ = 1.3 µM), melanoma (B16F10, IC_50_ = 6 µM), cervical (HeLa, IC_50_ = 5.5 µM), and breast carcinoma (EMT6/AR1, IC_50_ = 14.7 µM) cells. Notably, it showed minimal cytotoxicity to non-cancerous breast epithelial (MCF10A, IC_50_ = 23.2 µM) and fibroblast (L929, IC_50_ = 14 µM) cells. Mechanistically, compound **1** inhibits tubulin polymerization by binding to the colchicine site and altering tubulin secondary structure. This tubulin disruption led to apoptotic cell death through G2/M phase cell cycle arrest.

Building on previous work on promising anti-mitotic drugs targeting microtubules, Khan et al. reported a novel library of 1,4-dihydroindenopyrazole-oxindole conjugates and tested for their in vitro cancer inhibitory activity toward HeLa, A549, MDA-MB231, and HEK293 cell lines [34]. The antitumor evaluation results demonstrated that among all the tested compounds, the dimethoxy-containing analogs **2**, **3**, and **4**, with chlorine and methoxy substitutions at the 6-position of the e-ring, exhibited the strongest antitumor activity against tested cell lines (IC_50_ = 1.33–4.33 µM). Compound **4** demonstrated upregulated levels of the tumor suppressor proteins p53, p21, and the pro-apoptotic protein BAX, while compound **2** induced apoptosis through activation of caspase-3. These compounds inhibited tubulin polymerization by occupying the colchicine binding site on tubulin, and arrested the cell cycle in the G2/M phase.

Cui et al. designed and prepared a class of 1*H*-benzofuro[3,2-*c*]pyrazole derivatives to identify novel tubulin polymerization inhibitors [35]. The cancer cell growth inhibitory activity of all derivatives was screened against K562, A549, and MCF7 cells with ABT-751 as a standard. Among them, compound **5** displayed the most potent inhibitory activity, being much more active than the standard drug ABT-751. Additionally, the IC_50_ value of tubulin polymerization inhibition of compound **5** was 7.30 µM, identifying it as a new tubulin polymerization inhibitor.

Two series of 3,4-diaryl pyrazole derivatives were designed and synthesized based on the highly potent tubulin polymerization inhibitor CA-4 by Romagnoli et al. [36]. To explore their biological activity, all derivatives were screened for their anticancer activity against six cancer cell lines. Compound **6** showed the highest antitumor activity in all compounds with IC_50_ of 0.06–0.25 nM. Following in vivo experiments, compound **6** also displayed significant tumor growth inhibitory activity at low concentrations (5 mg/kg) in an orthotopic murine mammary tumor model. Compound **6** was one of the most potent tubulin polymerization inhibitors (IC_50_ = 0.35 µM), and a potent colchicine binding inhibitor, with 96% and 90% inhibition at the concentrations of 5 and 0.5 µM, respectively.

Wang et al. designed and synthesized novel benzimidazole-grafted benzene sulfonamide-pyrazole hybrids and evaluated their in vitro anticancer activity as potential tubulin polymerization inhibitors [37]. The research results revealed that compound **7** showed the highest antiproliferative activity toward A549, Hela, HepG2, and MCF7 (IC_50_ = 0.15–0.33 µM), using colchicine and CA-4 as positive controls. Compound **7** also displayed the most remarkable inhibitory activity of tubulin assembly with IC_50_ of 1.52 µM by binding to the colchicine site on tubulin. The inhibition mechanism of compound **7** on tubulin polymerization was similar to that of CA-4, occurring in a concentration-dependent manner. Additionally, compound **7** could arrest cell cycle in G2/M phase, induce cell apoptosis, and disrupt the cellular microtubule network.

A library of pyrazolo[1,5-*a*]pyrimidine analogs were reported by Li et al. as novel inhibition candidates of tubulin polymerization and screened for their antiproliferative activity on HeLa, MCF7, A549, HCT116, and B16F10 cancer cells with colchicine and paclitaxel as standard drugs [38]. The biological results revealed that compounds **8** and **9** exhibited the highest cytotoxicity (average IC_50_ = 24.8 nM and 28 nM) against all tested cell lines. Compounds **8** and **9** could inhibit in vitro tubulin polymerization in a dose dependent manner by binding to the colchicine site on tubulin (IC_50_ = 6.7 µM and 2.1 µM). Mechanism studies indicated both compounds arrested the cell cycle in G2/M phase, and inhibited cancer cell motility and migration. Moreover, compound **9** exhibited highly effective suppression of B16-F10 melanoma tumor growth in vivo without apparent toxicity. Therefore, compound **9** was identified as a promising tubulin inhibitor.

Wang et al. prepared a new class of pyrazole-naphthalene analogs and assessed for their in vitro antitumor activity against MCF7 cancer cells [39]. Following the results, analog **10** demonstrated the highest activity (IC_50_ = 2.78 µM), being five times more potent than the positive control cisplatin (IC_50_ = 15.24 µM). In addition, compound **10** showed excellent inhibition of tubulin polymerization (IC_50_ = 4.6 µM) compared to colchicine (IC_50_ = 6.7 µM), indicating it is a novel potent tubulin polymerization inhibitor. Compound **10** was able to stably bind to the colchicine-binding site on tubulin and induce cell cycle arrest at G2/M phase, leading to apoptosis.

Cherukumalli et al. prepared a library of novel substituted aryl urea derivatives of pyrimidine-pyrazole as potential antiproliferative agents against four human cancer cell lines (MCF7, A549, Colo205, and A2780) [40]. Among the synthesized derivatives, compound **11** revealed the strongest antiproliferative activity against all tested cell lines with IC_50_ values ranging from 0.01 to 0.65 µM, superior to the standard drug etoposide. Molecular docking studies demonstrated that compound **11** occupied the colchicine binding site, demonstrating it is a promising tubulin inhibitor.

Doan et al. designed and synthesized a series of 1-aryl-1*H*-pyrazole-fused curcumin analogs and evaluated their cytotoxicity against MDA-MB231 and HepG2 cancer cell lines using an MTT assay [41]. Among the analogs, compounds **12**, **13,** and **14** exhibited potent cytotoxicity against both cell lines, with IC_50_ values ranging from 3.64 to 16.13 µM. More hydrophobic structures in these compounds led to weaker cytotoxicity, possibly due to poor absorption and permeability through cell membranes. Moreover, compound **12**, **13,** and **14** exhibited excellent inhibitory activity of tubulin polymerization with IC_50_ values of 52.03, 45.29, and 40.76 µM, respectively. All three compounds were able to induce caspase-3 activity higher than control levels in MDA-MB231 at 10 µM after 48 h, indicating they can produce apoptosis in MDA-MB231 by increasing caspase-3 activity.

Sagam et al. synthesized novel morpholine-benzimidazole-pyrazole hybrids as potential tubulin polymerization inhibitors for cancer treatment [42]. In vitro antitumor screening against MCF7, PC3, and A549 human cancer cell lines showed that compound **15** had higher activity than the standard CA-4, with IC_50_ values of 0.042, 0.61, and 0.76 µM against MCF7, PC3, and A549 cells, respectively. This highlights compound **15** as a potent tubulin polymerization inhibitor warranting further development. Furthermore, compound **15** was three times more potent at inhibiting in vitro tubulin polymerization than CA-4, with an IC_50_ value of 0.35 µM. Molecular docking studies showed that compound **15** had the highest binding energy (−9.69 kcal/mol) and inhibition constant (78.75 nanomolar) with the α, β-tubulin. Therefore, compound **15** is a promising new tubulin polymerization inhibitor with potent antiproliferative activity.

Pyrene-pyrazole pharmacophore (PPP) was reported as the skeleton structure of microtubule targeted drugs by Sar et al. [43]. Using copper-catalyzed cross-dehydrogenative coupling, they synthesized a novel series of pyrene-pyrazole analogs. Biological experiments demonstrated that PPPs could regulate tubulin polymerization. Cytotoxicity assays of PPPs against MCF7, MDA-MB231, and C32 cells showed compound **16** had the highest inhibitory activity, with IC_50_ values of 1, 0.5, and 5.0 µM, respectively. Notably, compound **16** did not significantly impact the viability of normal MCF10A cells. The selectivity of PPPs for tumor over normal cells may be due to greater dysregulation of tubulin polymerization in cancer cells.

## 3. Kinase Inhibitors

Protein kinases play a fundamental role in regulating cell growth, differentiation, and apoptosis, and therefore serve as important therapeutic targets for developing anticancer agents. The development of small molecule inhibitors against these kinases represents an effective strategy for cancer treatment. The latest review has summarized 42 protein kinase inhibitors containing an unfused pyrazole ring that are in clinical testing stages, including inhibitors of AKT, EGFR/VEGFR, MAPK, BRAF, JAK, C-Met, BTK, etc. Representative drug candidates include afuesertib, lazertinib, dexmetinib, encorafenib, ruxolitinib, crizotinib, and pirobrutinib [44].

Beyond the clinically tested inhibitors, increasing efforts have focused on the development of novel pyrazole-based protein kinase inhibitors against the aforementioned molecular targets for precision oncology.

**Figure 2:**
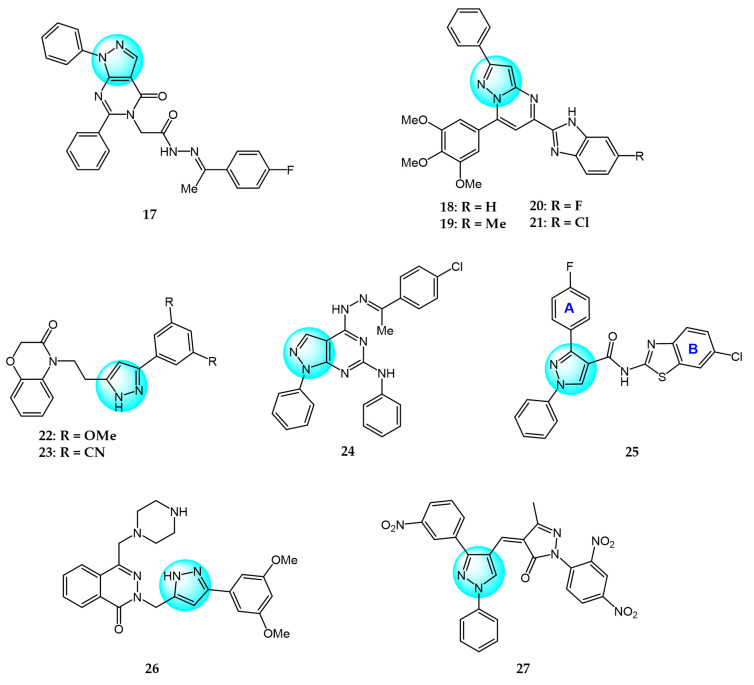
Pyrazole-containing kinase inhibitors targeting EGFR and VEGFR (compounds **17**–**27**).

For targeting EGFR, Gaber et al. synthesized several pyrazolo[3,4-*d*]pyrimidine derivatives based on the key pharmacophoric features of potent EGFR inhibitors [45]. These derivatives were evaluated for their cytotoxicity against MCF7. Among them, derivative **17** (Figure 2 for structures of compounds **17**–**27**) showed the highest inhibitory activity (IC_50_ = 2.89 µM) on MCF7, comparable to the standard drug toceranib (IC_50_ = 2.28 µM) and superior to doxorubicin (IC_50_ = 4.27 µM). Structure–activity relationship studies revealed that linking an aryl or heteroaryl moiety via a hydrophilic linker greatly improved anticancer activity. Molecular docking studies showed that compound **17** could bind to the active site of the EGFR kinase domain, suggesting it may act as a promising EGFR inhibitor.

Bagul et al. designed and synthesized a novel series of benzimidazole-linked pyrazolo[1,5-*a*]pyrimidines and evaluated their antiproliferative activity against various human cancer cell lines including MCF7, A549, HeLa, and SiHa [46]. Several compounds displayed potent and selective cytotoxicity. Notably, compounds **18**, **19**, **20,** and **21** exhibited strong inhibitory activity effects on the cancer cell lines with IC_50_ values in the micro- to nano-molar range, while showing minimal toxicities toward non-cancerous MRC5 cells. These results reveal these benzimidazole-pyrazolo[1,5-a]pyrimidine derivatives as promising anticancer agents warranting further investigation. And compound **20** with a 3,4,5-trimethoxyphenyl at C-7 and 5-F on the benzimidazole C-5 position was the most potent against MCF7, A549, HeLa, and SiHa with IC_50_ values of 3.2, 4.2, 8.9, and 7.9 µM, respectively. Immunostaining and Western blot analyses showed downregulation of EGFR, p-EGFR, STAT3, p-STAT3, and the anti-apoptotic protein Bcl-2 and procaspase-9, along with upregulation of pro-apoptotic proteins like p53, p21, and BAX, confirming these compounds could induce apoptosis. Compounds **18**, **19**, **20,** and **21** also exhibited excellent EGFR inhibitor activity with IC_50_ values of 0.82, 0.31, 0.37, and 0.29 µM, compared to the positive control erlotinib (IC_50_ = 0.45 µM).

Benarjee et al. synthesized a library of 3,5-disubtituted 1,4-benzoxazine-pyrazole hybrids and evaluated their in vitro antiproliferative activity against various cancer cell lines including MCF7, A549, HeLa, and PC3 [47]. Among the derivatives, compounds **22** and **23** showed potent cytotoxicity with IC_50_ values ranging from 2.82 to 6.28 μM, comparable to the standard drug etoposide. Screening for EGFR inhibitory activity revealed compounds **22** and **23** had the most promising activity (IC_50_ = 0.6124 and 0.5132 µM, respectively), superior to the standard erlotinib. Molecular docking study showed that compounds **22** and **23** possessed strong binding interactions with the target EGFR protein, where the binding energies are −8.61 and −10.36 Kcal/mol, respectively.

Gaber et al. reported a series of novel 1*H*-pyrazolo[3,4-d]pyrimidine derivatives as potential EGFR inhibitors [48]. These compounds were evaluated for their anticancer activity against non-small cell lung cancer A549 cells and colorectal carcinoma HCT116 cells. Among the derivatives, compound **24** demonstrated potent cytotoxicity against both cell lines with IC_50_ values of 8.21 and 19.56 µM, respectively. Furthermore, compound **24** exhibited significant EGFR inhibitory activity against wild-type EGFR and the resistant T790M mutant, with IC_50_ values of 0.016 and 0.236 µM, respectively. Structure–activity relationship analyses revealed that introducing aliphatic amines at the 4-position of the pyrazolo[3,4-*d*]pyrimidine scaffold attenuated cytotoxicity, while incorporation of an aniline moiety at this position enhanced potency. Mechanistic studies showed that compound **24** induced apoptosis and cell cycle arrest at S and G2/M phases, it also markedly upregulated BAX gene expression and downregulated Bcl-2 expression. In summary, compound **24** displays promising antiproliferative and pro-apoptotic activities mediated through EGFR inhibition.

VEGF and its receptor VEGFR-2 are critical regulators of endothelial cell proliferation and migration [49]. A series of pyrazole-based inhibitors targeting this critical pathway have been designed and developed in the laboratory. Reddy et al. synthesized a novel series of pyrazole benzothiazole hybrids as potential anticancer and antiangiogenic agents, followed by screening them against several cancer cell lines like HT29, PC3, A549, U87MG, and normal human cells HEK293T [50]. Compound **25** exhibited potent activity against all tested cancer cells with IC_50_ values ranging from 3.17 to 6.77 µM, superior to the reference drug axitinib. Structural–activity relationship analyses revealed that compounds with electron withdrawing groups on ring A or ring B showed the greatest growth inhibition. Notably, compound **25** displayed an IC_50_ value of 97 nM against VEGFR-2, comparable to the standard (IC_50_ = 39 nM). Mechanistically, compound **25** induces cell cycle arrest and apoptosis by depolarization of the mitochondrial membrane potential, increase in ROS, and damaging of DNA. Furthermore, it showed robust binding interactions with VEGFR-2 active sites and inhibited intersegmental blood vessel formation in transgenic zebrafish.

Badithapuram et al. prepared a library of phthalazine-piperazine-pyrazole conjugates and tested their antiproliferative activity against MCF7, A549, and DU145 cells [51]. Among them, conjugate **26** showed significant activity against all cancer cells tested, with IC_50_ values of 0.96, 1.40, and 2.16 µM for MCF7, A549, and DU145, respectively. This was superior to the standard drug etoposide. Compound **26** also showed low cytotoxicity against the normal breast cell line MCF10A. Structure–activity relationship studies indicated compound **26** exhibited more potent cytotoxicity with strong electron-donating methoxy substituents at the 3 and 5 positions of the phenyl ring. Additionally, compound **26** had higher inhibitory activity against VEGFR-2 tyrosine kinase (IC_50_ = 34.58 µM) compared to sorafenib. Docking study demonstrated that compound **26** showed more binding interactions with VEGFR-2 than VEGFR-1.

Dawood et al. prepared pyrazolone-pyrazole derivatives through a series of synthetic reactions and screened their in vitro inhibition on MCF7 [52]. The best inhibitory activity was revealed by compound **27** with an IC_50_ value of 16.50 µM compared to 23.31 µM for the standard drug tamoxifen. Some of these pyrazole derivatives reduced VEGFR-2 levels to varying degrees. Notably, compound **27** showed 78% inhibition (IC_50_ = 828.23 nM) of VEGFR-2, emerging as a promising anti-breast cancer drug candidate targeting this receptor. Further research demonstrated that compound **27** promoted pre-G1 apoptosis and inhibited G2/M phase growth by activating caspase-3, exhibiting significant pro-apoptotic activity.

**Figure 3:**
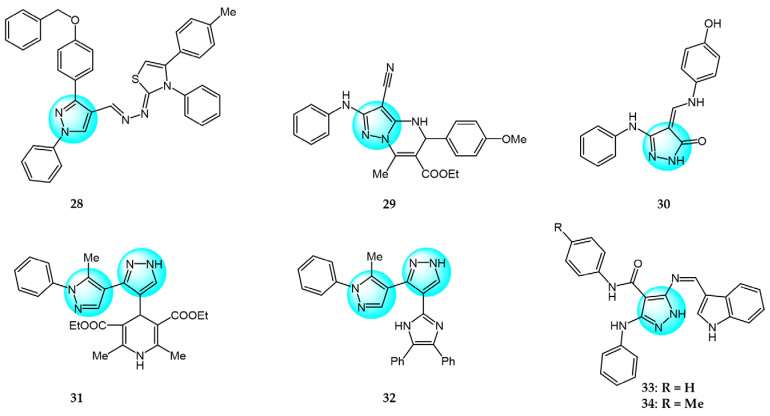

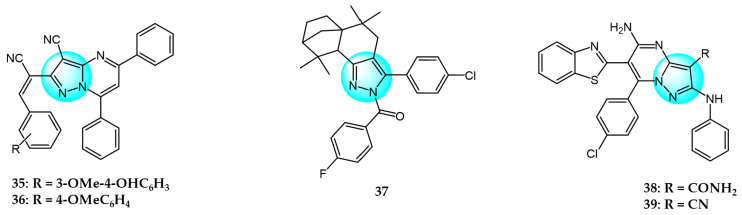
Pyrazole-containing kinase inhibitors targeting CDKs (compounds **28**–**39**).

Cyclin-dependent kinases (CDKs) are a family of serine-threonine kinases and play an important role in cell division, gene transcription, and other key biological processes [53]. Harras et al. reported several pyrazole derivatives possessing 1,3,4-trisubstituted and screened antitumor activity against different cancer cell lines, such as HCT116, UO31, and HepG2 [54]. Most derivatives exhibited potent cytotoxic activity, with compound **28** showing superior potency compared to the positive control sorafenib. Compound **28** had IC_50_ values of 0.035, 2.24, and 0.028 µM in HCT116, UO31, and HepG2 cells, respectively, with no clear toxicity toward normal cells. Additionally, compound **28** (Figure 3 for structures of compounds **28**–**39**) induced G2/M cell cycle arrest and overexpressed caspase-3, leading to apoptosis cell death in hepatocellular cancer cells. Analyses showed that compound **28** also possessed excellent CDK1 inhibitory activity with IC_50_ of 1.52 µM.

Ali et al. designed and synthesized different classes of novel pyrazole and pyrazolo [1,5-*a*]pyrimidine compounds as potential CDK2 inhibitors [55]. These compounds were evaluated for their antitumor activity on MCF7, HepG2, A549, and Caco2. The results revealed that compound **29** exhibited the highest cytotoxic activity, with IC_50_ values of 17.12, 10.05, 29.95, and 25.24 µM, respectively. Among the studied compounds, compound **30** displayed significant inhibitory activity against CDK2/cyclin A2 protein kinase at a concentration of 10 µM, with 60% inhibition.

Kuthyala et al. synthesized and evaluated a new series of pyrazole-based hybrid heteroaromatics for their in vitro antiproliferative activity against A549 lung cancer cells [56]. Among the derivatives, compounds **31** and **32** exhibited the most potent activity, with IC_50_ values of 42.79 and 55.73 μM, respectively against the A549 cells. They did not exhibit any toxicity to normal cell lines. Molecular docking confirmed that compounds **31** and **32** displayed effective binding with CDK2 protein with minimum binding energies of −5.372 and −7.676 Kcal/mol, respectively. Therefore, they were identified as potent inhibitors of CDK2 protein dysregulation in cancer cells.

Hassan et al. designed and developed some novel indole derivatives linked to the pyrazole moiety and screened them for in vitro antitumor activity against four human cancer cell lines (HCT116, MCF7, HepG2, and A549) [57]. Most of the synthesized compounds showed moderate to excellent cytotoxicity activity, with derivatives **33** and **34** displaying the most potent cancer inhibition (IC_50_ < 23.7 µM) which was even better than the standard reference drug doxorubicin (IC_50_ = 24.7–64.8 µM). In addition, compounds **33** and **34** revealed significant inhibitory activity toward CDK2 (IC_50_ = 0.074 and 0.095 µM). These two compounds induced significant downregulation of caspase-3 activities and Bcl-2 protein levels, along with upregulation of the BAX protein level, causing apoptosis. Molecular docking studies showed that compounds **33** and **34** could interact with CDK2 active pocket through different interactions. Therefore, these two compounds were considered to be promising CDK2 inhibitors and anticancer drug candidates.

Metwally et al. prepared a series of new pyrazolo[1,5-*a*]pyrimidine derivatives and evaluated their antitumor efficacy toward HepG2, MCF7, and Hela [58]. Among them, compound **35** exhibited the best inhibitory activity with IC_50_ values of 3.53, 6.71, and 5.16 µM for HepG2, MCF7, and Hela, respectively. Through an evaluation of CDK2 enzyme inhibition, compound **36** revealed the most significant inhibition against CDK2 with an IC_50_ value of 0.199 µM.

Wang et al. designed and synthesized a series of novel pyrazole ring-containing isolongifolanone derivatives as potential antitumor drugs and tested their antitumor activity on three cancer cell lines (MCF7, A549, and Hela) [59]. Compound **37** exhibited the most potent antiproliferation activity toward MCF7 with an IC_50_ value of 5.21 µM. Mechanism study demonstrated that compound **37** induced apoptosis through activation of caspase-3 and PARP, downregulation of Bcl-2, and upregulation of BAX and p53. In addition, compound **37** induced intracellular ROS generation and mitochondrial depolarization. It could bind to CDK2 by forming hydrogen bonds with amino acid residues and decrease the level of CDK2.

Khedr et al. synthesized a new class of benzothiazolyl pyrazolopyrimidine carboxamide and benzothiazolyl pyrazolopyrimidine carbonitrile derivatives and evaluated their cytotoxic activity against 60 cell lines [60]. Among the derivatives, compounds **38** and **39** displayed the highest cytotoxicity toward most of the cell lines tested. Molecular docking studies against CDK2 and CDK9 enzymes revealed that compound **38** exhibited the highest free energy of binding against CDK2 (−8.10 kcal/mol), while compound **39** exhibited the highest free energy of binding against CDK2 (−8.16 kcal/mol) and CDK9 (−7.87 kcal/mol). Furthermore, compound **39** was identified as the most potent inhibitor against CDK2 and CDK9 with IC_50_ values of 127 and 65 nM, respectively.

**Figure 4:**
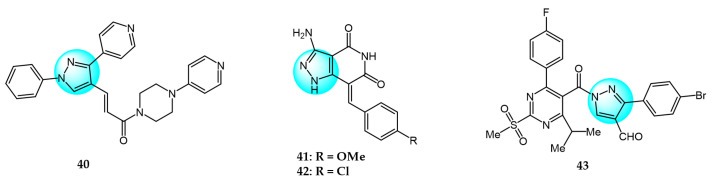
Pyrazole-containing kinase inhibitors targeting PI3K/AKT and MARK/ERK (compounds **40**–**43**).

PI3K/AKT and MAPK/ERK are crucial cancer signaling cascades. Inhibition or concurrent blockade of these two pivotal pathways represents a potential strategy for cancer therapy [61,62]. Demiroglu-Zergeroglu et al. studied the in vitro potential anticancer activity of a series of 1,3-diarylpyrazole acrylamide derivatives against mesothelial cells (MeT-5a), malignant mesothelioma (SPC212), and lung cancer cell lines (A549). Among all the synthesized derivatives, compound **40** (Figure 4 for structures of compounds **40**–**43**) revealed excellent antiproliferative activity [63]. Further evaluation showed that compound **40** could trigger caspase-dependent apoptosis in SPC212 cells and significantly inhibit the phosphorylation and expression of ERK1/2 and AKT proteins at concentrations above 10 µM in 24 h. In addition, compound **40** could induce G2/M cell arrest in a dose- and cell-dependent manner, with SPC212 cells being more susceptible than A549 cells. Therefore, compound **40** exhibited potent inhibitory activity on MAPK/ERK and PI3K/AKT pathways and was considered as a promising anticancer candidate.

Metwally et al. synthesized some novel pyrazolo[4,3-*c*]pyridine derivatives and tested their antitumor activity [64]. The antiproliferative assay showed that compound **41** demonstrated the most potent activity on MCF7 and HepG2 (IC_50_ = 1.937 and 3.695 µg/mL), while compound **42** showed excellent inhibitory activity against HCT116 with an IC_50_ value of 2.914 µg/mL compared to the standard drug doxorubicin (4.162, 3.832, and 3.676 µg/mL for MCF7, HepG2, and HCT116, respectively). In addition, docking studies showed that compound **41** binds to the inside of the active site of 5buj enzyme with similar and additional interactions compared to the reported natural ligands, indicating these compounds may act as ERK2 inhibitors.

Thangarasu et al. [65] designed and synthesized a series of novel pyrazole carbaldehyde derivatives as potential PI3 kinase inhibitors for anticancer drug development. The compounds were evaluated for their anti-breast cancer and anti-inflammatory activities. Among the derivatives, compound **43** was identified as the most potent PI3 kinase inhibitor. It exhibited excellent cytotoxicity against MCF7 breast cancer cells with an IC_50_ of 0.25 μM compared to 0.95 μM for the standard drug doxorubicin. These results demonstrate that compound **43** is a promising lead candidate for further optimization as a targeted breast cancer therapy through its potent and selective inhibition of PI3 kinase signaling.

**Figure 5:**
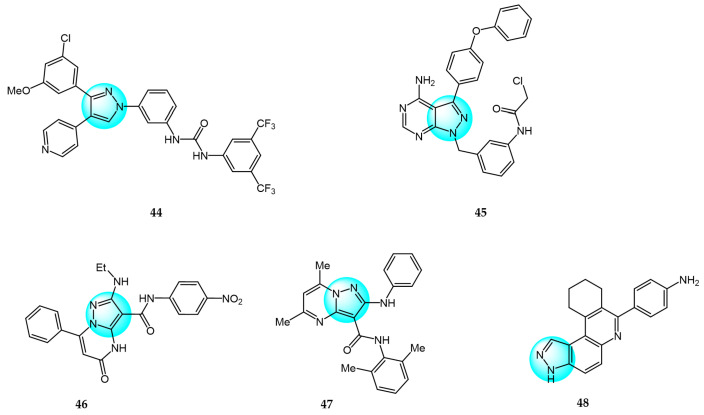
Pyrazole-containing kinase inhibitors targeting BRAF^V600E^, BTK, PIM-1, and haspin kinase (compounds **44**–**48**).

Compared to pyrazole-based inhibitors targeting common tumor-associated targets like EGFR and VEGFR, the development of pyrazole-based BRAF inhibitors has been relatively limited thus far, likely due to challenges such as resistance [66]. Encorafenib is one of the few reported pyrazole-cored agents used to target BRAF [44]. Beyond encorafenib, scientists continue to synthesize and optimize other pyrazole derivatives targeting BRAF, with the goal of achieving enhanced selectivity and antitumor efficacy. Therefore, El-Gamal et al. designed and synthesized 20 pyrazole-containing diarylureas and diarylamides and evaluated their antiproliferative activity against a panel of 58 cancer cell lines [67]. Among the compounds, derivative **44** (Figure 5 for structures of compounds **44**–**48**) displayed the strongest broad-spectrum anticancer activity with sub-micromolar IC_50_ values, showing superior potency compared to the reference diarylurea drug sorafenib. Compound **44** was extremely selective against cancer cells compared to normal cells, with an IC_50_ value above 100 µM for RAW 264.7 macrophages. Furthermore, compound **44** exhibited high activity and selectivity against V600E-BRAF kinase with an IC_50_ value of 0.39 µM, indicating this is an antiproliferation mechanism at molecular level. Another mechanism underlying the antiproliferative activity of compound **44** was inducing apoptosis through stimulating caspase-3/7 activity (EC_50_ = 1.52 µM for RPMI-8226).

Bruton’s tyrosine kinase (BTK) is an important target in B cell malignancies. It plays a key role in B cell receptor signaling involved in B cell functions. Ibrutinib is the first FDA approved BTK inhibitor for cancer therapy and often used as a positive control when designing new BTK inhibitors [68]. Ran et al. designed and synthesized a series of pyrazolopyrimidine derivatives as BTK inhibitors and evaluated their antitumor activity against mantle cell lymphoma (MCL) cell lines [69]. Among the compounds, derivative **45** exhibited the most potent and effective antitumor activity with sub-micromolar IC_50_ values across multiple MCL cell lines including Mino, Jeko-1, Z138, and Maver-1. Compound **45** also showed strong inhibition of BTK with an IC_50_ value of 27 nM, which was slightly less potent than the reference BTK inhibitor ibrutinib (IC_50_ = 8 nM). Moreover, compound **45** displayed higher selectivity against BTK and more effective antiproliferative activity in primary patient tumor cells compared to ibrutinib. Moreover, it could strongly induce apoptosis in Jeko-1 and Z138 cells at low micromolar concentrations.

PIM-1 is an important oncogenic protein kinase, whose overexpression can lead to various cancers [70]. The first-generation oral PIM inhibitor SGI-1776 containing a pyrazole core structure can inhibit the activity of PIM kinases, and has shown anticancer activity in preclinical models of lung cancer and prostate cancer. This verifies the strategic significance of using pyrazole compounds to design PIM-1 inhibitors, but most of these compounds are still at the laboratory research stage, and preclinical evaluations of their anticancer efficacy and safety need to be further completed. For example, Philoppes et al. synthesized some new pyrazolo[1,5-*a*]pyrimidine derivatives as PIM-1 inhibitors and tested their antitumor activity toward HCT116 and MCF7 cells [71]. Among the compounds, derivatives **46** and **47** revealed the most potent antiproliferative activity. Compound **46** inhibited HCT116 cells with an IC_50_ value of 1.51 μM. Meanwhile, compound **47** was most active against MCF7 cells with an IC_50_ value of 7.68 μM. Therefore, both compounds were evaluated for their PIM-1 inhibitory activity and the test results showed that compounds **46** and **47** exhibited remarkable inhibitory activity (IC_50_ = 0.60 and 0.67 µM, respectively) compared to the reference drug vemurafenib (IC_50_ = 0.31 µM). The research shows that high hydrophobic ligands had better bonding affinity since most of the lining residues in previously reported inhibitors are lipophilic, which requires hydrophobic interactions. Compound **47** exhibited the highest hydrophobic character which could well explain its optimal antitumor activity against breast cancer cells.

Haspin is a serine/threonine kinase whose overexpression or absence can lead to mitotic defects, making it a promising anticancer target. Compared to other anticancer drugs, haspin inhibitors have shown potent anticancer effects with fewer side effects. However, only a few haspin inhibitors have been reported to date [72]. To develop pyrazole-based haspin inhibitors, Opoku-Temeng et al. studied the inhibitory effects of a series of pyrazolo[4,3-*f*]quinoline derivatives on haspin kinase across different cancer cell lines [73]. Among all the compounds, compound **48** exhibited the highest growth inhibition at 10 μM, with IC_50_ values of 1.7 and 3.6 μM in HCT116 and HeLa cells, respectively. In vitro assays showed compound **48** inhibited over 90% of haspin enzyme activity at a concentration of 100 nM, identifying it as a potent haspin kinase inhibitor.

## 4. Multitargeted Kinase Inhibitors

Multitargeted kinase inhibitors can simultaneously inhibit multiple tumor signaling pathways, reducing the likelihood of drug resistance and exhibiting great potential for precision cancer therapy. As the pioneering multi-kinase inhibitor, sorafenib targets key receptors including VEGFR, PDGFR, and KIT, achieving dual blockade of angiogenesis and proliferation. It has been established as the standard first-line therapy for advanced hepatocellular carcinoma and renal cell carcinoma. The introduction of sorafenib has heralded a new era of multitargeted agents for cancer, paving the way for next-generation multi-kinase inhibitor development. It is the preferred first-line treatment for many metastatic solid tumors and commonly employed as a control arm in clinical trials. While long-term use inevitably confers resistance, sorafenib’s success has spearheaded the adoption of multitargeted combinatorial regimens. This also represents a pivotal opportunity for further breakthroughs in precision oncology [74]. 

The recent review has reported three unfused pyrazole-containing multi-kinase inhibitors currently in clinical trials: crizotinib, merestinib, and ilorasertib [44]. Additionally, extensive efforts have been made to develop novel multitargeted kinase inhibitors incorporating pyrazole core scaffold for precision oncology. These agents may confer advantages over traditional inhibitors by enhancing target specificity and minimizing off-target effects. Further studies are warranted to fully evaluate their therapeutic potential for precise malignancy treatment.

**Figure 6:**
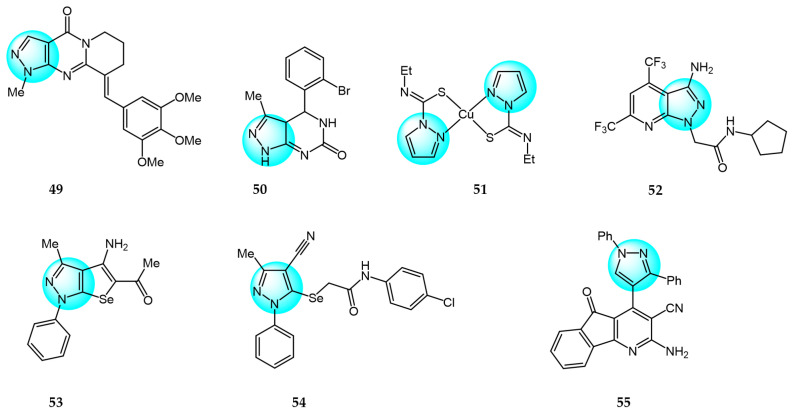
Pyrazole-containing multitargeted kinase inhibitors (compounds **49**–**55**).

Recently, Ruzi et al. prepared several pyrazolo[3,4-*d*]pyrimidine derivatives bearing carbon-aryl(heteryl)idene moieties as potential anticancer agents [75]. The highest antiproliferative activity in vitro was exhibited by compound **49** (IC_50_ = 0.03–6.561 µM) against 15 cancer cell lines (Figure 6 for structures of compounds **49**–**55**). It also showed in vivo cytotoxicity in xenograft HT-29 tumor-bearing mice and induced G2/M phase arrest in cancer cells by decreasing the mitochondrial membrane potential. In addition, compound **49** inhibited VEGFR-2 kinase activity by forming hydrogen bonds with cys-919 and exhibited inhibitory activity on tubulin polymerization by binding to the colchicine-binding site on tubulin. Therefore, the anti-vascular effects and inhibition of tubulin polymerization are the main sources of anticancer activity of compound **49**.

Saleh et al. synthesized a series of different fused pyrazole derivatives and evaluated their in vitro inhibitory activity against human tumor cell lines HepG2, EGFR, and VEGFR-2 [76]. The results revealed that compound **50** demonstrated potent dual EGFR and VEGFR-2 inhibition (IC_50_ = 0.09 and 0.23 µM) and it displayed more excellent cytotoxicity against HepG2 (IC_50_ = 0.71 µM) than the standard drugs erlotinib (10.6 µM) and sorafenib (1.06 µM).

Ghorbanpour et al. synthesized a series of bidentate nitrogen and sulfur donor pyrazole-based ligands and evaluated their antitumor activity against MCF7 breast cancer cells using the MTT assay [77]. Compared to the free ligands, Cu (II) complexes showed significant cytotoxicity and improved interactions with EGFR and CDK2 proteins. Among the synthesized ligands, compound **51** demonstrated the highest cytotoxicity against MCF7 with an IC_50_ value of 20.70 µM, which is much lower than other investigated ligands.

Bhukya et al. designed and synthesized a series of novel pyrazolo[3,4-*b*]pyridine amide/amino acid functionalized derivatives and screened their in vitro antiproliferative activity against four cancer cell lines A549, MCF7, DU145, and HeLa [78]. Among the tested derivatives, compound **52** exhibited significant activity with IC_50_ values of 21.2, 18.4, 19.2, and 25.3 µM against A549, MCF7, DU145, and HeLa, respectively, compared to the reference standard 5-fluorouracil. Moreover, compound **52** displayed strong binding affinity toward EGFR and HER2 receptors with the lowest binding energy of -10.98 kcal/mol and inhibition constant of 23.91 µM.

Zaki et al. reported some 5-alkylated selanyl-1*H*-pyrazole derivatives and their 4-amino-5-substituted selenolo[2,3-*c*]pyrazole analogs as potential anticancer agents [79]. These analogs were evaluated for their anticancer activity against HepG2 cell line. The results revealed that most of the synthesized compounds displayed potent inhibition at low concentrations against the cancer cell line. Compounds **53** and **54** showed the highest activity (IC_50_ = 15.98 and 13.85 µM). Additionally, both were found to be potent dual inhibitors of EGFR and VEGFR-2, which could explain their superior anticancer properties.

Nossier et al. reported some new 1,3,4-triarylpyrazoles containing different heterocycles and tested their cytotoxic activity against human cancer cell lines (HepG2, MCF7, PC3, A549, and HCT116) [80]. Among them, compound **55** exhibited the highest anticancer activity on MCF7, A549, and HCT116 (IC_50_ = 6.53, 26.40 µM, and 59.84 µM, respectively), compared to doxorubicin which had IC_50_ values of 45.0, 48.8, and 65.1 µM against these cells. Furthermore, compound **55** was evaluated for its in vitro inhibition of twelve protein kinases. The results revealed potent inhibition (over 94%) of six kinases (AKT1, AKT2, BRAF V600E, EGFR, p38α, PDGFRβ), especially 99% inhibition of EGFR. Four other kinases (VEGFR-2, CDK2/Cyclin A1, and both of the PI3 kinases) showed moderate inhibitions ranging from 47% to 76%.

## 5. Inhibitors of Other Targets

### 5.1. DNA Binding Agents

DNA binding inhibitors are a class of small molecule compounds that inhibit tumor cell proliferation by interacting with DNA. Compared with traditional cytotoxic chemotherapy drugs, DNA binding inhibitors have the advantages of high selectivity, unique mechanism of action, low side effects, and ability to overcome drug resistance. This provides new strategies and options for cancer treatment [81].

**Figure 7:**
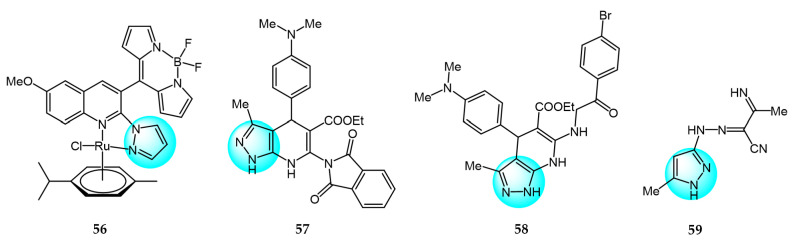
Pyrazole-containing inhibitors as DNA binding agents (compounds **56**–**59**).

Paitandi et al. designed several novel arene ruthenium complexes and tested for in vitro antiproliferative and photocytotoxic activities against HeLa human cervical cancer cells [82]. Among the compounds, complex **56** (Figure 7 for structures of compounds **56**–**59**) exhibited the highest cytotoxicity when exposed to visible light (IC_50_ = 12.87 µM) and low dark toxicity (IC_50_ > 100 µM). Molecular docking studies showed that compound **56** binds to the minor groove of CT-DNA via van der Waals forces and electrostatic interactions. In addition, compound **56** demonstrated high singlet oxygen generation and could induce apoptosis in HeLa cells.

El-Gohary et al. synthesized novel pyrazolo[3,4-*b*]pyridine analogs and screened them for in vitro anticancer activities against HepG2, MCF7, and HeLa cancer cell lines [83]. Among the compounds, **57** and **58** showed remarkable cytotoxicity toward HepG2, MCF7, and HeLa cells (IC_50_ = 3.11–4.91 and 4.06–4.24 µM, respectively), compared to the reference drug doxorubicin (DOX) (IC_50_ = 4.30–5.17 µM). Notably, compounds **57** and **58** exhibited lower toxicity than DOX in normal WISH and W138 cells. DNA binding assays revealed that compounds **57** and **58** had strong DNA binding affinities (IC_50_ = 27.13 and 29.15 µM, respectively) and appeared to interact with DNA similarly to doxorubicin.

A novel series of polysubstituted pyrazole derivatives were synthesized by Omran et al. and screened for their antitumor activity against HepG2 hepatocellular carcinoma cells [84]. Among the compounds, **59** exhibited the highest anticancer activity with an IC_50_ value of 2 µM, which was lower than the standard drug cisplatin (IC_50_ = 5.5 µM). Compound **59** demonstrated superior DNA binding affinity, displacing 90.14% of methyl green in a competitive assay. Molecular docking studies further showed that compound **59** could bind to the minor groove of DNA. Collectively, these results indicate that compound **59** is a promising anticancer agent that could potentially be combined with conventional chemotherapy regimens.

### 5.2. Topoisomerase Inhibitors

DNA topoisomerases, particularly type IIA, are considered ideal therapeutic targets for cancer treatment. They help relieve overwinding or underwinding of the DNA double helix during replication to maintain the topological stability of DNA. Studies have revealed that type IIA expression and activity are often upregulated several folds in tumor cells compared to normal cells. Thus, selectively inhibiting or downregulating type IIA activity in tumor cells could induce cancer cell death while minimizing toxicity to normal cells [85]. 

**Figure 8:**
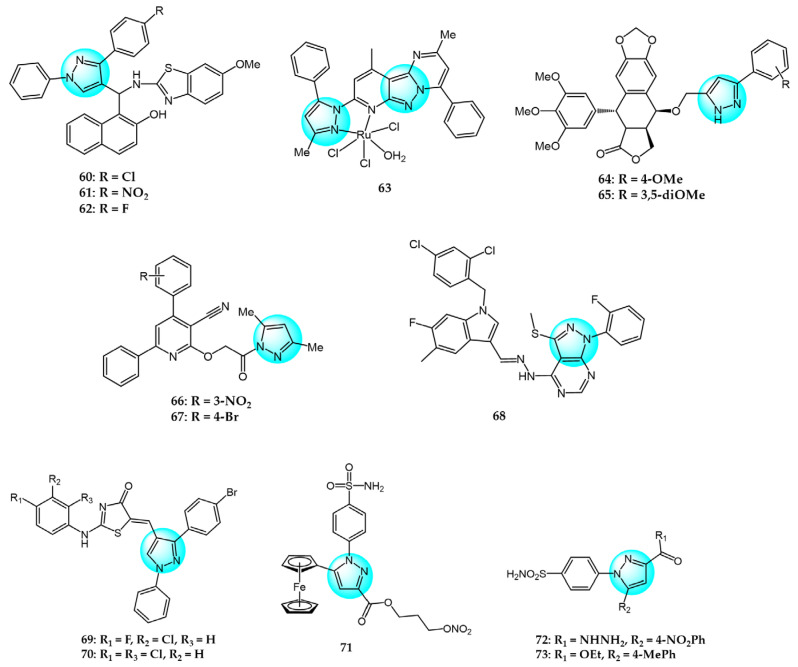
Pyrazole-containing inhibitors targeting topoisomerase, Eg5, MDM2, COX-2, and hCA IX isoenzyme (compounds **60**–**73**).

Nagaraju et al. designed and prepared novel pyrazole-linked benzothiazole-β-naphthol derivatives as topoisomerase I inhibitors [86]. The derivatives were screened for their cytotoxicity against A549, HeLa, and MCF7 cells using the MTT assay. Most compounds exhibited significant cytotoxicity, especially **60**, **61**, and **62** (Figure 8 for structures of compounds **60**–**73**), with IC_50_ values of 4.63–5.54 µM. These compounds did not significantly affect normal HEK293 cell growth (IC_50_ > 45 µM). Flow cytometry showed the derivatives arrested the cell cycle at G2/M phase. Molecular docking demonstrated that they bind to the DNA minor groove with high affinity and effectively inhibited topoisomerase I.

Gu et al. prepared three ruthenium(III) complexes with pyrazolopyrimidine ligands and evaluated their antiproliferative activity against six cancer cell lines using the MTT assay [87]. Complex **63** exhibited potent antitumor activity against all cells tested (IC_50_ = 9.7–21.2 µM). It showed lower cytotoxicity in normal HL-7702 cells (IC_50_ = 20.0 µM) than cisplatin (IC_50_ = 19.6 µM). Complex **63** induced apoptosis by upregulating cytochrome C, BAX, p53, and Apaf-1 and downregulating Bcl-2 through elevating intracellular ROS and Ca^2+^ and reducing mitochondrial membrane potential. It arrested the cell cycle at S phase by reducing CDC25, cyclin A2, and CDK2 expression. Additionally, complex **63** interacted with DNA and inhibited topoisomerase I, causing DNA damage.

Nagavath et al. synthesized novel 4β-aryl pyrazole-epipodophyllotoxin derivatives as topoisomerase II inhibitors [88]. Compounds **64** and **65** exhibited superior anticancer activity versus the standard drug against five cancer cell lines with IC_50_ values ranging from 1 to 100 nM. Both displayed strong interactions with topoisomerase II, with binding energies of −12.11 and −12.51 kcal/mol and inhibitory concentrations of 675.96 pM and 1.34 nM, respectively. Thus, compounds **64** and **65** were promising topoisomerase II-targeting anticancer agents.

Zebbiche et al. synthesized novel pyrazole derivatives and evaluated their cytotoxic activity in Caco2 and MCF7 cells [89]. At 0.1 and 1 µM, respectively, compounds **66** and **67** showed greater cytotoxicity against Caco2 cells than the docetaxel standard, with logIC_50_ values of −0.657 and −0.498 µM. Both compounds displayed strong affinity for the topoisomerase-IIβ active site, contributing to their cytotoxicity.

### 5.3. Eg5 Inhibitors

Kinesin-5 (Eg5), also known as kinesin family member 11 (KIF11), is one of 45 different motor protein superfamilies in the human genome. It is widely expressed in normal tissues and plays a key role in spindle microtubule assembly, chromosome arrangement, and separation. Eg5 is an attractive antitumor drug target, and its inhibitors have been extensively studied for cancer chemotherapy [90].

Muthuraja et al. investigated a class of pyrazolopyrimidine derivatives as potent Eg5 inhibitors and evaluated their in vitro antitumor activity against HeLa cells [91]. According to the test result, compound **68** exhibited the most potent inhibitory activity with an IC_50_ value of 1.43 µM, inhibiting Eg5 at micromolar concentration (IC_50_ = 16.42 µM). Molecular docking studies revealed that compound **68** strongly interacted with residues Trp127 and Arg119, conferring high binding affinity for Eg5 active site 1.

### 5.4. MDM2 Inhibitors

MDM2 is an important negative regulator of the tumor suppressor gene p53. Overexpression of MDM2 can bind to p53 and inhibit its transcriptional activation activity, thereby promoting tumorigenesis. Therefore, designing small molecule inhibitors targeting the MDM2–p53 interaction can effectively reactivate the p53 pathway and exert antitumor effects [92]. 

Bhat et al. designed and synthesized a novel series of thiazolidinone-pyrazole hybrids and in vitro and in vivo anticancer activities were evaluated against Ehrlich ascites carcinoma (EAC) and MDA-MB231 cells [93]. Compound **69** demonstrated excellent cytotoxicity against EAC (IC_50_ = 901.3 µM), which was superior to the standard drug (IC_50_ = 1954.4 µM). Molecular docking studies showed compound **69** was strongly docked to MDM2 protein with the best docking score of −36.75 kJ/m, indicating it is a potent MDM2 inhibitor. Additionally, compound **70** exhibited the highest inhibitory activity against MDA-MB231 with an IC_50_ value of 29.8 µM, highlighting the importance of dichloro substituents on the arylamino thiazolidinone nucleus for enhancing potency.

### 5.5. COX-2 Inhibitors

Cyclooxygenase-2 (COX-2) has long been considered a target for pain relief and treating inflammation. However, it is also expressed in many cancers, where it promotes apoptotic resistance, proliferation, angiogenesis, inflammation, invasion, and metastasis [94].

Ren et al. synthesized novel ferrocene-pyrazole derivatives containing nitric oxide donors and evaluated their anticancer activities against Hela cells [95]. Compound **71** exhibited the most potent activity (IC_50_ = 0.34 µM) and significantly inhibited COX-2 (IC_50_ = 0.28 μM), compared to celecoxib (IC_50_ = 0.38 and 7.91 µM, respectively). Further studies showed compound **71** induced Hela cell apoptosis through mitochondrial depolarization and G1 cell cycle arrest in a dose- and time-dependent manner. Moreover, compound **71** markedly increased ROS levels, leading to oxidative DNA damage. It also generated the highest nitric oxide concentration, potently inhibiting Hela cell proliferation. Testing of PGE2 production from arachidonic acid revealed compound **71** reduced PGE2 levels, implicating it as a COX-2 inhibitor acting through the COX-2/PGE2 pathway.

### 5.6. hCA IX Isoenzyme Inhibitors

hCA IX is a carbonic anhydrase isoenzyme that is overexpressed in many tumor cells. The major role of hCA IX is to catalyze the conversion of carbon dioxide and water to bicarbonate, thereby maintaining extracellular pH homeostasis. This is crucial for the survival of tumor cells. Therefore, it is considered an important target for the design of antitumor drugs. Yamali et al. synthesized a series of 1,3,5-trisubstituted pyrazoles carrying benzene sulfonamides and evaluated their inhibitory activity against the tumor-associated hCA IX isoform, HSC-2 carcinoma cell line, and normal HGF cells [96]. The results showed that derivative **72** displayed superior inhibition and selectivity for the hCA IX isoenzyme (Ki = 2.3 nM) while exhibiting low nanomolar inhibitory potency against hCA II (Ki = 8.9 nM). Therefore, compound **72** was considered a promising anticancer drug candidate for further research and development of CAIs. However, cytotoxicity assays revealed that compound **73** exhibited more potent antitumor activity with a CC_50_ value of 37.7 µM compared to the reference drugs 5-FU and MTX.

## 6. Pyrazole Derivatives with Undefined Mechanisms

Although numerous pyrazole derivatives have been rationally designed and developed with well-characterized anticancer mechanisms, a substantial number of reported pyrazole derivatives demonstrate varying degrees of anticancer activity but with poorly defined mechanisms of action. These compounds exhibit anticancer effects. However, their precise modes of action and putative cellular targets warrant further in-depth investigation. Elucidating the anticancer mechanisms of these pyrazole-based compounds would not only facilitate comprehension of their mechanisms of action and structure–activity relationship studies, but also enable the development of novel highly selective targeted anticancer therapeutics. 

**Figure 9:**
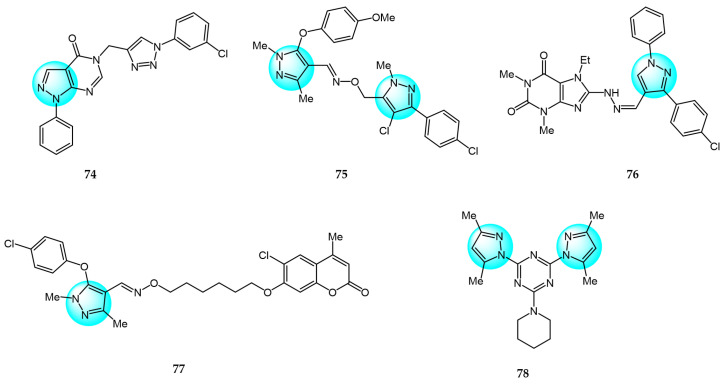

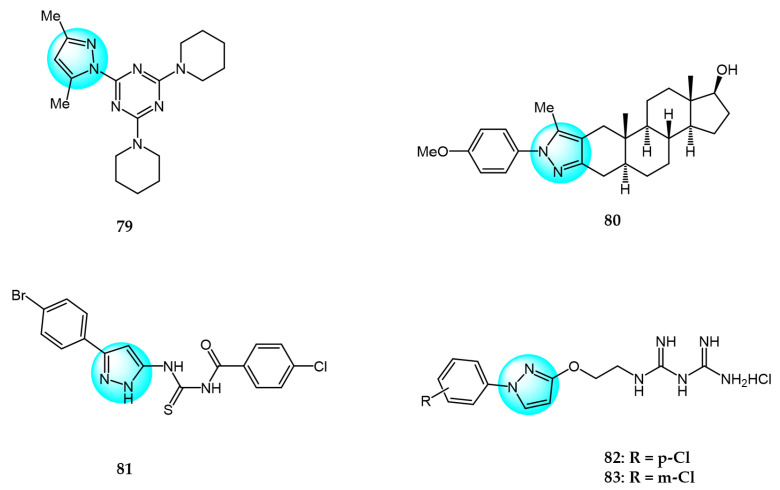
Pyrazole derivatives with anticancer activity (compounds **74**–**83**).

Allam et al. synthesized a series of hybrid aza-heterocycles containing pyrazolo[3,4-*d*]pyrimidin-4(5*H*)-ones conjugated to a 1,2,3-triazole scaffold and evaluated their antitumor efficacy against C6 and U87 cell lines in vitro [97]. Following screening, compound **74** (Figure 9 for structures of compounds **74**–**83**) exhibited the strongest inhibitory effect with 17.87% and 47.69% growth inhibition against C6 and U87 cells, respectively. Further studies revealed that compound **74** could induce S phase cell cycle arrest and apoptosis in U87 cells by triggering caspase-3 and PARP cleavage. Additionally, compound **74** was found to strongly interact with the TGFBR2 protein.

In another study, Dai et al. designed, synthesized, and evaluated the anticancer activity of a series of bis-pyrazole derivatives across three human cancer cell lines (SMMC7721, SGC7901, and HCT116) [98]. Among the derivatives, compound **75** displayed the most potent effect with IC_50_ values ranging from 0.76 to 2.01 µM, superior to the standard drugs 5-FU and ADM, without impacting non-tumor liver cells. It also exhibited excellent inhibition of hepatoma tumors in vivo with low toxicity in mice. Moreover, compound **75** dose-dependently induced apoptosis by upregulating BAX expression, downregulating Bcl-2 expression, and promoting PARP and caspase-3 cleavage. Further analyses demonstrated that the anticancer activities of compound **75** may be related to DNA damage and activation of the p53 signaling pathway.

Shaaban et al. designed and synthesized a series of novel 8-substitued purines incorporating a pyrazole moiety and evaluated their antitumor activity against five human cancer cell lines, including A549, MCF7, HepG2, Caco2, and PC3 [99]. Among the compounds, compound **76** exhibited potent and selective inhibitory activity (IC_50_ = 18.89, 169.20, 244.95, 732.26, and 201.01 µM), compared to the standard anticancer drug 5-FU. Moreover, compound **76** induced higher levels of apoptosis than 5-FU in all cell lines tested, evidenced by caspase-3/7 activation and DNA damage. 

Dai et al. synthesized a series of coumarin/pyrazole oxime derivatives and evaluated their anticancer activity against SMMC-7721 cells [100]. Among the derivatives, compound **77** displayed the most potent anticancer activity with an IC_50_ value of 2.08 µM, compared to 5-FU (IC_50_ = 37.8 µM) and ADM (IC_50_ = 2.67 µM), and low cytotoxicity toward normal LO2 cells. Additionally, compound **77** significantly inhibited metastasis by reducing migration and suppressing EMT-related protein expression, including E-cadherin, vimentin, and MMP9.

Farooq et al. reported a series of mono- and di-pyrazolyl-s-triazine derivatives, and evaluated their activity against four human cancer cell lines: Breast carcinoma (MCF7 and MDA-MB231), hepatocellular carcinoma (HepG2), colorectal carcinoma (LoVo), and leukemia (K562) [101]. Compounds **78** and **79**, containing a piperidine moiety, were most effective at inhibiting cell survival across all cell lines tested, with IC_50_ values ranging from 5 to 21.2 µM. The cytotoxic mechanism was suggested to involve the induction of S and G2/M phase cell cycle arrest. 

Mótyán et al. synthesized novel 17-keto steroidal pyrazole analogs and examined their antiproliferative activity against cervical (HeLa), breast (MCF7 and MDA-MB231), and prostate (PC3 and DU145) carcinoma cells [102]. Among the derivatives, compound **80** displayed the highest cancer cell specificity with IC_50_ values ranging from 3.6 to 8.5 µM, compared to cisplatin which showed IC_50_ values ranging from 126.8 to 237.3 µM.

Nitulescu et al. prepared 1*H*-pyrazol-5-ylthiourea and (1,5-dimethyl-3-oxo-2-phenyl-pyrazol-4-yl)thiourea derivatives using ultrasound-mediated synthesis, and evaluated their cytotoxicity against HT29 and THP1 cells [103]. Compound **81** exhibited potent inhibitory activity against THP1 with IC_50_ of 40.34 µM, and induced G2/M arrest by upregulating cell cycle-related genes.

Zhou et al. reported pyrazole-containing biguanide derivatives and examined their cytotoxicity against bladder (UMUC3, T24) and ovarian (A2780) cancer cells [104]. Compounds **82** and **83** displayed potent activity, with IC_50_ values ranging from 6.9 to 23.4 µM, and significantly improved over metformin and phenformin. Mechanistic studies revealed compounds **82** and **83** could activate AMPK and inhibit mTOR signaling.

**Figure 10:**
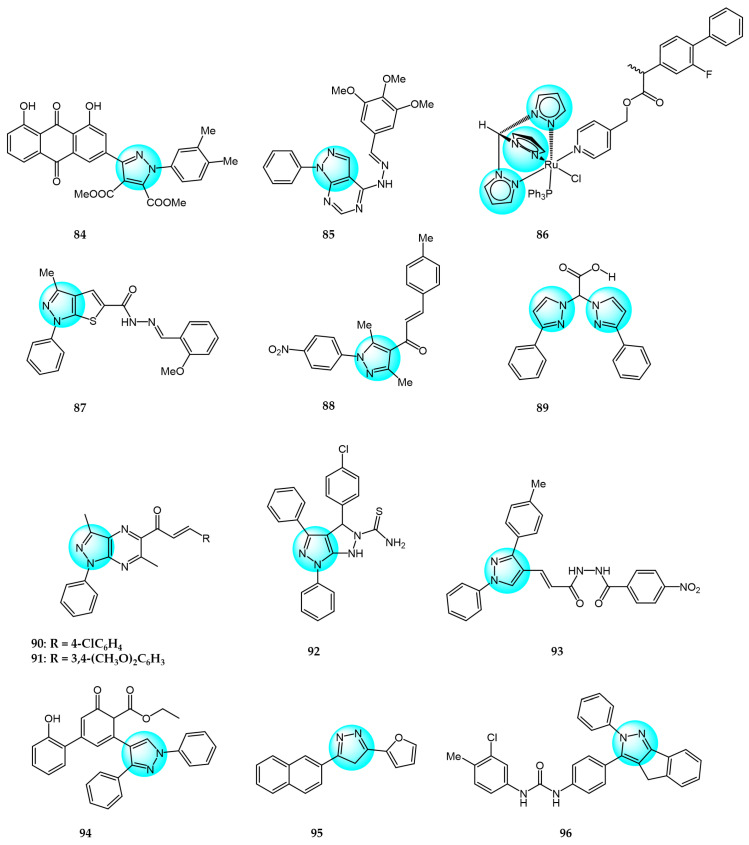

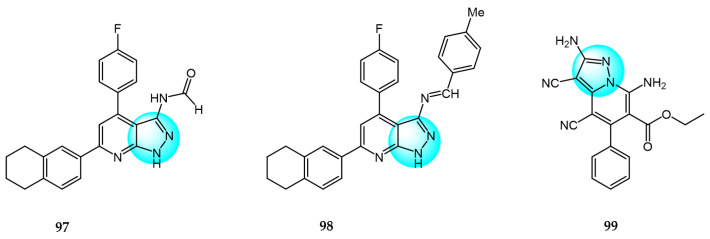
Pyrazole derivatives with anticancer activity (compounds **84**–**99**).

Kumar et al. synthesized aloe-emodin derivatives and evaluated their anticancer activity in vitro against a panel of cancer cell lines including MDA-MB231, MCF7, HepG2, and B16F10 [105]. Most derivatives displayed improved potency compared to aloe-emodin. Notably, compound **84** (Figure 10 for structures of compounds **84**–**99**) exhibited remarkable activity with IC_50_ values of 1.32 and 0.99 µM against MDA-MB231 and MCF7 cells, respectively, compared to doxorubicin (IC_50_ = 2.56 and 2.02 µM). Compound **84** was also less toxic to normal cells. Mechanistic studies revealed compound **84** could induce G2/M cell cycle arrest and activate initiator and executioner caspases in MDA-MB231 cells. 

Othman et al. reported novel purine bioisosteres and examined their anticancer activity against Caco2, A549, HT1080, and Hela cells [106]. Among the compounds, compound **85** displayed the highest potency with IC_50_ values ranging from 17.50 to 73.08 µM. Moreover, the derivatives showed no significant toxicity in vivo. While compound **85** did not induce apoptosis in A549 cells, it significantly decreased Ki67 expression.

Gobbo et al. synthesized ruthenium(II) tris(pyrazolyl)methane complexes and evaluated their antiproliferative activity against A2780, A2780cisR, and non-tumoral HEK 293T cells, using cisplatin and RAPTA-C as positive and negative controls, respectively [107]. The complexes displayed cytotoxicity toward all cell lines tested, indicating a lack of cancer cell selectivity. Among the derivatives, complex **86** exhibited the highest activity with IC_50_ values of 4.5 and 8 μM against A2780 and A2780cisR cells, respectively. Enzyme inhibition assays suggested the antitumor activity were not closely associated with COX-2 and GSTP1 inhibition.

Hess et al. reported a unique thieno[2,3-*c*]pyrazole derivative **87** and evaluated its in vitro anticancer activity against a panel of 17 human cancer cell lines [108]. Compound **87** exhibited excellent potency against 14 cell lines with CC_50_ values less than 1 µM. Mechanistic studies revealed that compound **87** interfered with cell cycle progression, disrupted microtubule and mitotic spindle formation, reduced phosphorylation of p38, CREB, Akt, and STAT3 kinases, and induced hyperphosphorylation of FGR, HCK, and ERK1/2 kinases. Apoptosis was identified as the primary mechanism of 87-induced HL-60 cell death, mediated through caspase-3/7 activation, reactive oxygen species accumulation, and loss of mitochondrial integrity. 

Kamel et al. prepared and characterized new pyrazolyl-chalcone derivatives [109]. Compound **88** displayed the highest in vitro cytotoxicity against A549 and HepG2 cells with IC_50_ values of 44.3 and 57.9 µg/mL, respectively. Compound **88** significantly decreased the expression of ISL1, MALL, ASNS, and ACLY genes, as well as increased DNA damage and DNA fragmentation in cancer cells.

Adeniyi et al. computationally evaluated nitrogen-chelating ligands containing bis-pyrazole, bipyridine, and phenanthroline for inhibitory anticancer activity, and validated select compounds in vitro against HT29 cancer and KMST normal cells [110]. Among the ligands, compound **89** exhibited the highest potency against HT29 cells (IC_50_ < 6.25 mM) with no toxicity toward normal KMST cells (IC_50_ > 50 µM). Docking studies also predicted strong interactions of compound **89** with cancer-related receptors. 

El-Kashef et al. synthesized novel pyrazolo[3,4-*b*]pyrazine derivatives and tested for their in vitro anticancer activity against MCF7 cells [111]. The 4-Cl derivative **90** and 3,4-dimethoxy derivative **91** displayed the highest inhibitory activity with IC_50_ values of 2.29 and 2.22 µM, respectively, compared to paclitaxel (IC_50_ = 1.02 µM).

Nassar et al. reported the synthesis of pyrazolo[3,4-c]pyrazole derivatives and in vitro cytotoxicity evaluation against MCF7 cells [112]. All derivatives exhibited dose-dependent inhibition, with compound **92** displaying the highest potency (IC_50_ = 10.7 µM) compared to doxorubicin. These results suggest compound **92** could be developed into a more effective anticancer agent than doxorubicin.

Verma et al. synthesized pyrazolacrylic oxadiazole and amide derivatives and evaluated their antiproliferative activity against HCT116, SW620, HT29, and MCF7 cell lines [113]. Compound **93** exhibited good broad-spectrum inhibitory activity with IC_50_ values ranging from 1.8 to 4.4 µM, comparable to paclitaxel and camptothecin (IC_50_ < 0.8 µM).

Abdelgawad et al. synthesized novel substituted chalconated pyrazole analogs and tested their in vitro cytotoxicity against MCF7 and HepG2 cancer cells using MTT assay [114]. Compound **94** displayed the highest cytotoxic activity (IC_50_ = 6.93 and 7.38 µM), comparable to doxorubicin (IC_50_ = 4.50 and 4.17 µM). The introduction of a cyclohexan-2,4-dien-1-one ring and ester group contributed to the remarkable anticancer activity of compound **94**. 

Ahmed et al. reported novel pyrazole, oxazole, and pyridine analogs containing naphthalene and furan motifs, and evaluated their antitumor activity against HepG2 and MCF7 cells [115]. The pyrazole derivative **95** exhibited promising inhibition of HepG2 (IC_50_ = 17.14 µM) and MCF7 (IC_50_ = 9.76 µM) compared to doxorubicin (IC_50_ = 4.50 and 4.17 µM). Replacement of the hydrogen atom at N-1 of the pyrazole with COCH_3_ or CHO drastically reduced the inhibitory activity.

Gezegen et al. synthesized a library of indenopyrazole derivatives and evaluated their inhibition of HeLa and C6 glioma cells [116]. Compared to 5-FU (IC_50_ = 8.30 and 7.73 µM), compound **96** displayed superior inhibitory activity against HeLa (IC_50_ = 6.53 µM) and C6 cells (IC_50_ = 7.09 µM).

Hamza et al. synthesized novel tetralin-pyrazolo[3,4-*b*]pyridine hybrids and tested their in vitro cytotoxicity against HCT116 and MCF7 cells using MTT assay [117]. Compound **97** displayed the highest inhibition of MCF7 cells with an IC_50_ value of 16.1 µM, while compound **98** demonstrated the most potent anticancer activity against HCT116 cells with an IC_50_ value of 6.4 µM, compared to doxorubicin (IC_50_ = 9.5 and 65.6 µM, respectively).

A novel series of pyrazol [1,5-*a*]pyridine derivatives were prepared and evaluated for their antiproliferative activity against 60 human cancer cell lines by Naik et al. at the National Cancer Institute (NCI) [118]. In vitro anticancer assays showed that derivative **99** exhibited remarkable broad-spectrum inhibitory activity against most tested cell lines with GI_50_ values ranging from 1.18 to 8.18 µM. These results demonstrate that the C5-pyrazolo[1,5-*a*]pyridine scaffold represents a promising new class of anticancer agents with favorable drug-like properties.

**Figure 11:**
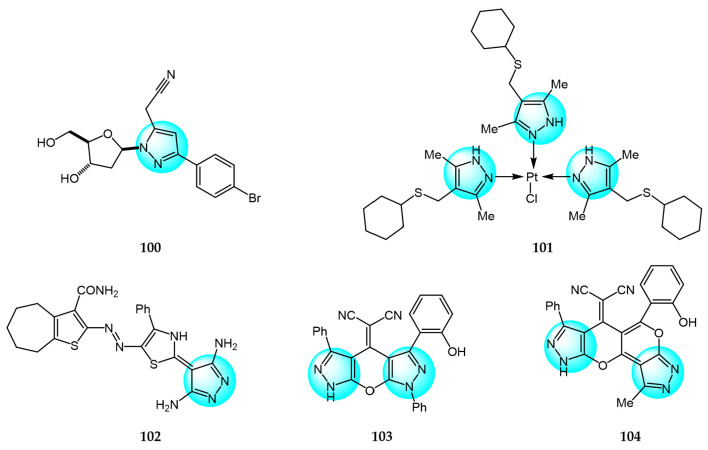

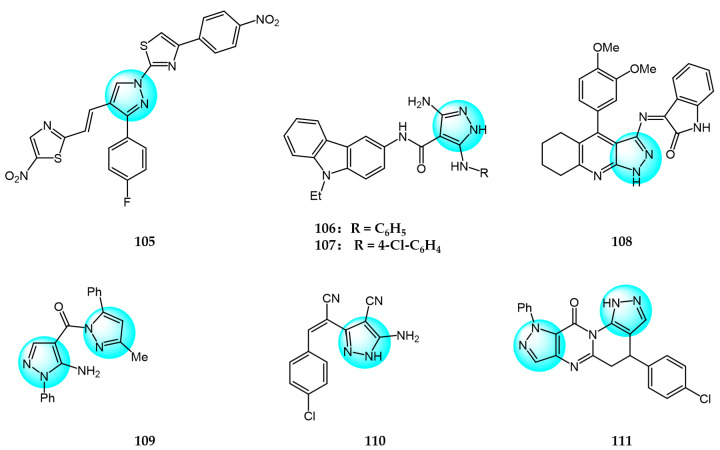
Pyrazole derivatives with anticancer activity (compounds **100**–**111**).

Yadav et al. prepared several novel pyrazolyl nucleoside derivatives and evaluated their anticancer and toxicity activity against the NCI-60 human cancer cell panel [119]. Biological evaluation results revealed that compound **100** (Figure 11 for structures of compounds **100**–**111**) showed moderate growth inhibition in 39 cell lines with GI_50_ values of 9.3 and 3.0 µM for Hop92 and HS 578T cells, respectively, without any observable toxicity even at the highest tested concentration.

Akhmetova et al. reported some novel mononuclear bi-ligand Pd(II) and tri-ligand Pt(II) complexes and evaluated their in vitro cytotoxic activity against Jurkat, K562, and U937 cancer cells and normal HEK293 cells [120]. The platinum complex **101** exhibited superior inhibitory activity (IC_50_ = 0.02, 0.09, and 0.10 µM for Jurkat, K562, and U937 cells, respectively) compared to cisplatin. 

Al-Ghorbani et al. synthesized several pyrazoles containing thiophene moieties and assessed their antitumor activity against HepG2, HCT116, MCF7, and PC3 cancer cells [121]. Compound **102** showed comparable or better cytotoxicity (IC_50_ values of 3.81, 5.85, 4.92, and 9.70 µM for HepG2, HCT116, MCF7, and PC3, respectively), versus doxorubicin (IC_50_ = 4.50, 5.23, 4.17, and 8.87 µM).

Bakhotmah et al. reported the synthesis and in vitro antiproliferative activity of tri- and tetracyclic pyrano[2,3-*c*]pyrazole derivatives against HCT116, HepG2, and MCF7 cells [122]. Compounds **103** and **104** displayed the most potent cytotoxicity against HCT116 (IC_50_ = 5.23 and 4.13 µM), HepG2 (IC_50_ = 6.17 and 5.29 µM), and MCF7 (IC_50_ = 8.27 and 6.79 µM) due to the phenyl or pyran rings and optimal linker. These results demonstrated the key role of the unique pyranopyrazole scaffold in conferring cytotoxicity.

Bansal et al. designed and synthesized thiazole-pyrazole conjugates [123]. In vitro anticancer assays showed compound **105** effectively inhibited MCF7 and HeLa cells with GI_50_ of 30.7 and less than 10 µg/mL, respectively, versus adriamycin (GI_50_ <10 µg/mL). Compound **105** also exhibited high activity (GI_50_ = 64.924 µg/mL) in inducing apoptosis of goat testicular cells at 10 µM.

Bondock et al. prepared several newly functionalized pyrazolo[3,4-d][1,2,3]triazin-4-ones and evaluated their antiproliferative activity against MCF7, HCT116, and HepG2 cells in vitro [124]. Compounds **106** and **107** showed potent activity against HCT116 (IC_50_ = 3.36 and 2.97 µM), HepG2 (IC_50_ = 3.90 and 3.37 µM), and MCF7 (IC_50_ = 8.53 and 8.77 µM), without toxicity to normal RPE1 cells.

Fathy et al. prepared new tetrahydroquinoline-pyrazole-hydrazide derivatives and assayed their in vitro anticancer activity against HepG2 and A549 cells [125]. The highest inhibitory activity against HepG2 and A549 was demonstrated by compound **108** (IC_50_ = 1.1 and 1.37 µM). This demonstrates that compound **108** may be a potential anticancer drug.

Hassan et al. synthesized novel pyrazole derivatives and evaluated their in vitro anticancer activity against various human cancer cell lines using adriamycin as positive control [126]. Compound **109** exhibited potent activity against MCF7 breast cancer cells (GI = 49.88%) and moderate activity against T-47D cells (GI = 38.15%), while compound **110** displayed strong anticancer activity against UO-31 renal cancer cells (GI = 42.81%). Moreover, compound **111** showed strong inhibition of CCRF-CEM leukemia (GI = 41.37%) and SR leukemia cells (GI = 44.95%).

**Figure 12:**
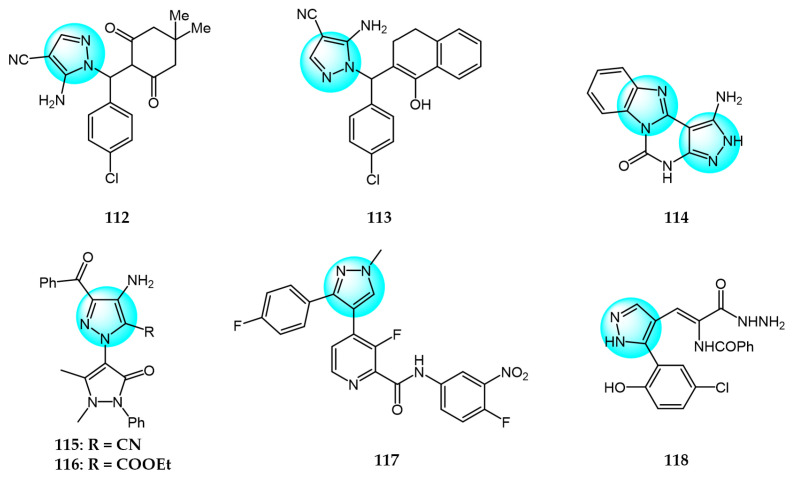

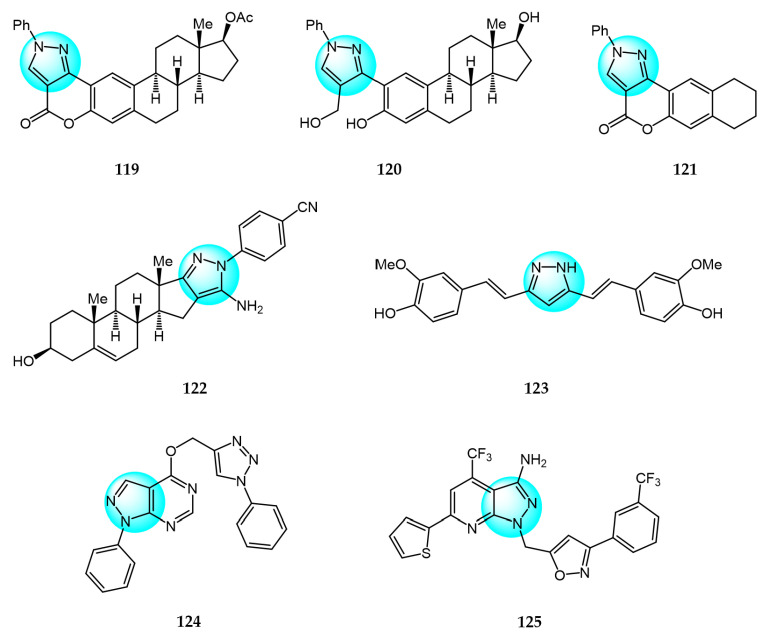
Pyrazole derivatives with anticancer activity (compounds **112**–**125**).

Hassan et al. synthesized fused pyrazolopyrimidines as pyrazoloquinazolines and benzoimidazo pyrazolopyrimidines, and tested them for antitumor activity on 60 human cancer cell lines [127]. Among the derivatives, compounds **112**, **113,** and **114** (Figure 12 for structures of compounds **112**–**125**) exhibited moderate to potent anticancer activities against certain cell lines. Compounds **112** and **113** showed higher COX-2 receptor affinity (−7.86 kcal/mol) than meclofenamine (−5.78 kcal/mol), conferring selectivity for COX.

Ismail et al. reported a novel class of hybrids, including pyrazolinylpyrazoles and pyrazolinyltriazoles, and screened them for antitumor activity on the NCI-60 human tumor cell line panel [128]. Compounds **115** and **116** exhibited remarkable potency against most of the tested cancer cell lines, with mean GI_50_ values of 5.47 and 2.24 µM, respectively. The broad-spectrum antitumor activities of compounds **115** and **116** against the NCI-60 panel highlight the pyrazolinylpyrazole and pyrazolinyltriazole scaffolds as promising new chemotypes for anticancer drug discovery.

Kankala et al. synthesized a series of fluorophenylpyrazole-picolinamide analogs and evaluated their in vitro inhibitory activity against four human tumor cell lines: HeLa (cervical), A549 (lung), MCF7 (breast), and IMR32 (neuroblastoma) [129]. Among the derivatives, compound **117** displayed the highest activity against HeLa, A549, MCF7, and IMR32 (IC_50_ = 4.25, 3.44, 2.63, and 8.99 µM, respectively), in comparison with the positive control cisplatin (IC_50_ = 4.98, 3.55, 1.56, and 9.23 µM, respectively).

Several new heterocycles containing chromone and pyrazole moieties were designed and prepared by Salem et al. [130]. The in vitro cytotoxic activity against HCT116 and MCF7 cancer cells were evaluated by MTT assay. The best inhibition against HCT116 and MCF7 was demonstrated by compound **118**, with IC_50_ values of 8.34 and 4.98 µg/mL, respectively, compared to the control drug doxorubicin. The enhanced cytotoxicity of compound **118** was attributed to its multiple active centers (OH, NH_2_, and 3NH groups).

Molnár et al. reported a novel series of A-ring-connected 2-pyrazoles of estradiol and evaluated their in vitro cytotoxicity against MCF7, PC3, DU145, and HeLa cancer cells [131]. Compounds **119**, **120,** and **121** exhibited remarkable inhibitory activities against HeLa, PC3, and DU145 (IC_50_ = 1.55, 2.90, and 1.41 µM, respectively).

Mótyán et al. synthesized novel D-ring-condensed 5-amino-1-arylpyrazoles and tested them for in vitro antiproliferative effects on human cancer cell lines including HeLa, U2Os, MCF7, PC3, and A549 [132]. Among the derivatives, compound **122** displayed significant cytotoxic activity against the tested cell lines with IC_50_ values ranging from 3.5 to 7.9 µM.

Pham et al. prepared 20 curcuminoids and pyrazole-modified curcuminoids and evaluated their antiproliferation against the HepG2 cancer cell line [133]. Compound **123** exhibited the most potent anticancer activity against HepG2 with an IC_50_ value of 1.53 μM. Structure–activity relationship analyses revealed that the OH group of the curcumin/pyrazole curcumin scaffold was an important factor for the antitumor activity.

Kalavadiya et al. synthesized a class of 1,2,3-triazole containing pyrazolopyrimidine derivatives and screened them for in vitro anticancer activity against 60 cell lines [134]. Compound **124** demonstrated good activity against the breast cancer cells T-47D.

Ravula et al. prepared novel isoxazole functionalized pyrazolo[3,4-b]pyridine derivatives and assayed them for their in vitro anticancer activity against cervical (HeLa), colon (COLO 205), liver (HepG2), and breast (MCF7) cancer cell lines [135]. Among the derivatives, compound **125** exhibited the most potent activity (IC_50_ = 11.3, 14.6, 13.4, and 9.1 µM, respectively).

**Figure 13:**
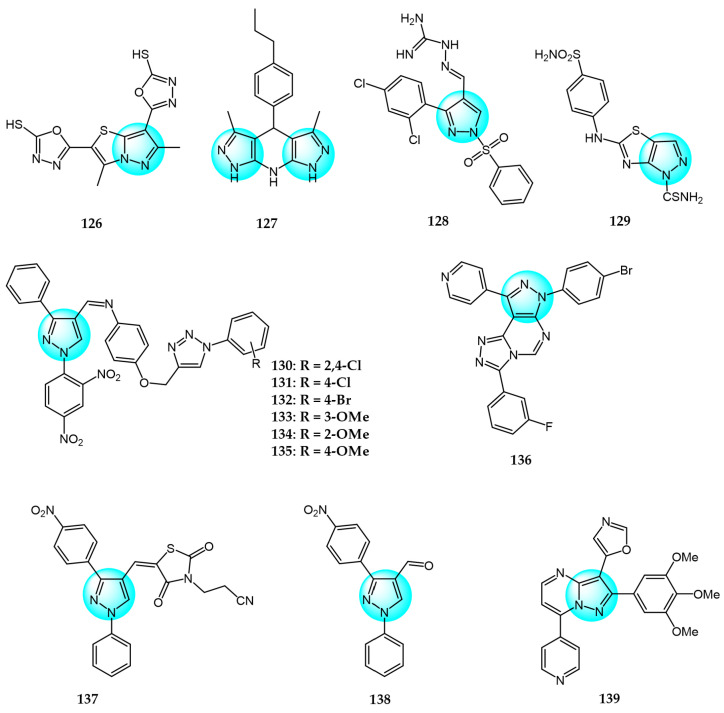
Pyrazole derivatives with anticancer activity (compounds **126**–**139**).

Alsayari et al. synthesized new pyrazolo[5,1-b]thiazole-based heterocycles and evaluated their in vitro inhibition against HepG2 hepatocellular carcinoma and HCT116 colon carcinoma cell lines using the MTT assay [136]. Compound **126** (Figure 13 for structures of compounds **126**–**139**) displayed that the best IC_50_ values were 6.9 and 13.6 µg/mL against both cell lines.

Chinthaparthi et al. synthesized tetrahydrodipyrazolo-pyridine derivatives and evaluated their in vitro antiproliferative effects on SK-BR3 and HeLa cancer cells [137]. Compound **127** demonstrated the most potent antitumor activity with IC_50_ values of 19.38 and 10.70 µM against HeLa and SK-BR3, respectively. The hydrophobicity and binding ability of the core scaffolds were attributed to their antiproliferative effects.

Huang et al. designed and synthesized 3-phenyl-1-phenylsulfonyl pyrazoles containing an aminoguanidine moiety and screened them for their in vitro antitumor activity against A549 and HeLa cell lines [138]. Compound **128** displayed promising inhibitory activity against A549 (IC_50_ = 1.90 µM) and HeLa (IC_50_ = 4.75 µM) compared to 5-fluorouracil (IC_50_ = 2.94 and 5.72 µM, respectively). In addition, it showed lower activity against 293T (IC_50_ = 41.72 µM).

Othman et al. synthesized pyrazolothiazole and thiazolopyridine analogs and evaluated their cytotoxicity against HepG2 hepatocellular carcinoma and MCF7 breast cancer cells [139]. Compound **129** exhibited the most potent inhibition against both cell lines with IC_50_ values of 10.89 and 15.60 µM, respectively, compared to 5-FU (IC_50_ = 26.75 and 32.75 µM).

Suryanarayana et al. prepared and characterized a novel library of dinitrophenylpyrazole bearing triazole scaffolds and evaluated their antiproliferation activity in vitro against MCF7, HeLa, and Caco2 carcinoma cells using combretastatin A4 as a reference drug (IC_50_ = 10, 9, and 11 µM, respectively) [140]. Compounds **130**, **131,** and **132** displayed potent activity against Caco2 with IC_50_ values ranging from 9 to 10 µM. Furthermore, compounds **133**, **134,** and **135** exhibited significant growth inhibitory activity against HeLa with IC_50_ values ranging from 4 to 6 µM. Notably, compound **135** was also highly potent against MCF7 with an IC_50_ value of 8 µM. 

Aliwaini et al. synthesized and studied three new pyrazolo[1,2,4]triazolopyrimidine derivatives and evaluated their cytotoxicity against cervical (HeLa) and breast (HCC1937 and MCF7) cancer cells [141]. Among them, compound **136** was revealed to be the most effective with IC_50_ value of 7.01 µM for HCC1937, 28.21 µM for MCF7, and 11.20 µM for HeLa. Further studies revealed that compound **136** could inhibit the activation of EGFR, protein kinase B (Akt), and extracellular signal-regulated kinase (ERK)1/2 by binding to the ATP binding site of EGFR, providing new insights for the development of EGFR-targeting drugs.

Alshammari et al. synthesized a new pyrazole-tethered thiazolidine-2,4-dione derivative and investigated the intermediate and final compounds for cytotoxicity against SW480 and MCF7 cell lines [142]. Compounds **137** and **138** exhibited potent inhibitory activity against SW480 (IC_50_ = 190 and 170 µg/mL, respectively) and MCF7 (IC_50_ = 90 and 140 µg/mL, respectively).

Bhogireddy et al. designed and synthesized substituted aryl derivatives of isoxazole-pyrazolo[1,5-*a*]pyrimidines and evaluated their anticancer activity toward PC3, DU145, A549, and MCF7 cancer cell lines [143]. Among these, compound **139** displayed the most remarkable antitumor activity (IC_50_ values: PC3 = 0.03 µM; A549 = 0.011 µM; MCF7 = 0.017 µM; and DU145 = 0.042 µM).

**Figure 14:**
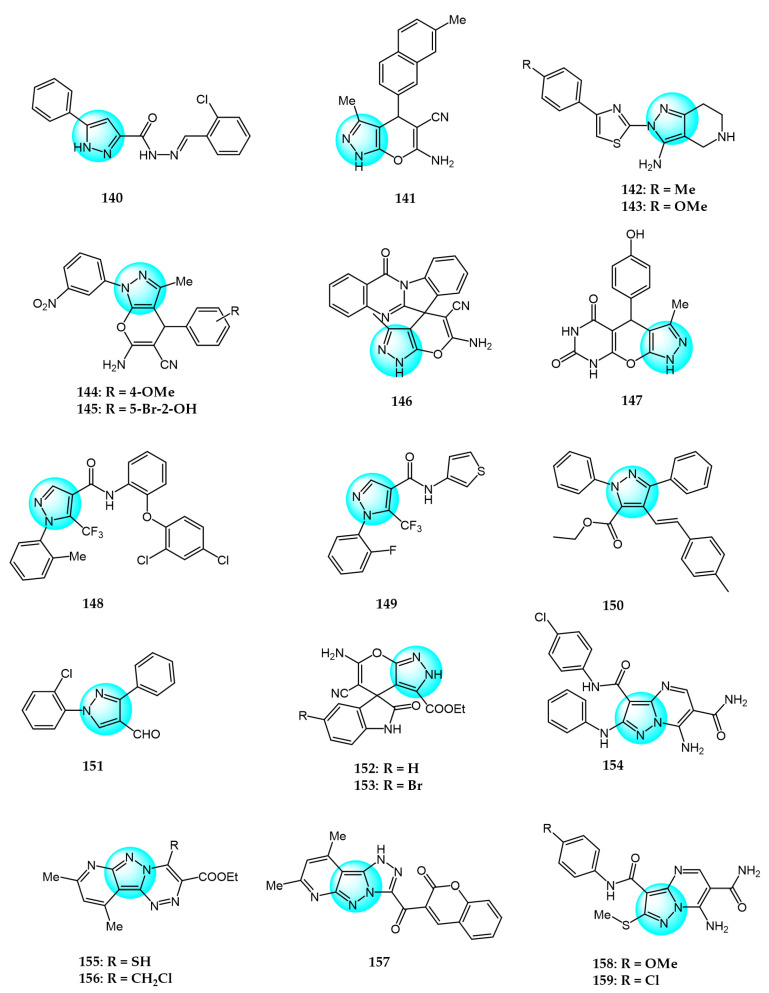
Pyrazole derivatives with anticancer activity (compounds **140**–**159**).

Karrouchi et al. synthesized three *N*’-arylidene-5-phenyl-1*H*-pyrazole-3-carbohydrazide derivatives and evaluated their in vitro cytotoxicity toward a panel of cancer cell lines including breast (BT474, MDA-MB231, AU565), chronic myeloid leukemia (K562), and lung (H460) cells [144]. Among these, compound **140** (Figure 14 for structures of compounds **140**–**159**) exhibited significant anticancer activity against H460 with an IC_50_ value of 0.15 µM using doxorubicin as reference (IC_50_ = 0.20 µM). 

Kumar et al. prepared a series of pyrano[2,3-c]pyrazoles via Knoevenagel condensation and Michael addition and evaluated their in vitro anticancer activity against PC3 and SKOV3 cells using an MTT assay [145]. All synthesized compounds displayed good to moderate activity, with compound **141** exhibiting the highest inhibitory activity with IC_50_ values of 7.5 µM and 6.9 µM against PC3 and SKOV3, respectively.

Several novel thiazolyl pyrazoles were synthesized and antitumor activity on HeLa, A549, and MDA-MB231 were evaluated by Mamidala et al. [146]. Compounds **142** and **143** demonstrated remarkable antiproliferation against HeLa (IC_50_ = 3.60 and 4.61 µM), A549 (IC_50_ = 4.17 and 5.29 µM), and MDA-MB231 (IC_50_ = 3.94 and 4.92 µM), respectively.

Parikh et al. synthesized and studied several new pyrano[2,3-c]-pyrazole-5-carbonitriles and evaluated their cytotoxicity toward several carcinoma cell lines including 786-0 (renal), A431 (epidermal), MCF7 (breast), and U251 (glioblastoma) using a CCK-8 assay [147]. Among these, derivative **144** exhibited good activity against 786-0 and MCF7 (IC_50_ = 9.9 and 31.87 µM), while compound **145** showed moderate activity against A431 and U-251 (IC_50_ = 19.98 and 25.78 µM).

Sadeghian et al. prepared a library of pyrano[2,3-c]pyrazole derivatives and determined their anticancer activity against breast (MDA-MB231), colon (HT29) cancer cells, and normal human dermal fibroblasts (HDF) using an MTT assay [148]. No compound displayed lower IC_50_ values against the tested cancer cells compared to the control drug etoposide. However, compound **146** exhibited significant cytotoxic activity against Panc1 with an IC_50_ value of 21.41 µM, lower than that of etoposide (IC_50_ = 24.35 µM).

Saleh et al. synthesized novel pyrazolopyranopyrimidine derivatives and assessed their antitumor properties against MCF7 breast cancer cells using an MTT assay [149]. Compounds containing barbituric acid and hydroxy groups showed the highest cytotoxicity, especially with the hydroxy group at the 4-position on the benzene ring (compound **147**). Thus, compound **147** exhibited the highest inhibitory activity against MCF7 with an IC_50_ value of 145 µM/mL.

Xie et al. designed and prepared 25 new 5-trifluoromethyl-1*H*-pyrazole-4-carboxamide derivatives and evaluated their antitumor activity against A549, PC3, and K562 cancer cell lines [150]. Among these, compound **148** (IC_50_ = 10.2 µM) exhibited greater anticancer activity than the commercial anticancer drug 5-fluorouracil (IC_50_ = 23.1 μM) against A549. Meanwhile, compound **149** (IC_50_ = 12.4 µM) showed enhanced anticancer activity compared to Gefitinib (IC_50_ = 22.2 µM) against PC3.

Xu et al. prepared and characterized some new fully substituted pyrazoles and evaluated their growth inhibitory activity against Huh7 cells [151]. Among these, compound **150** demonstrated excellent cell growth inhibition even at different concentrations compared to doxorubicin.

Otaibi et al. synthesized four novel hybrid compounds bearing pyrazole and chalcone moieties and evaluated their ability to inhibit the in vitro proliferation of human lung (A549) and colon (Caco2) cancer cell lines [152]. Following biological evaluation, the intermediate compound **151** exhibited greater cytotoxic activity than the final compounds against both cell lines, with increased sensitivity toward the A549 line.

Asif et al. reported some novel spirooxindole-pyranopyrazole derivatives and evaluated their in vitro anticancer activity against 60 human cancer cell lines at the National Cancer Institute (NCI) [153]. Compound **152** showed the highest 82% growth inhibition of the colon cancer cell line KM12 at 10 µM. Meanwhile, compound **153** inhibited the lung cancer cell line HOP92 with 43.19% growth inhibition.

Azher et al. synthesized a library of substituted pyrazolopyrimidine analogs and tested their in vitro antitumor activity against several cell lines including MCF7, PC3, Hep2, and WI38 [154]. Among these, compound **154** displayed significant potency against Hep2 and MCF7 with IC_50_ values of 8.85 and 10.80 µM, respectively, compared to the positive control 5-fluorouracil (IC_50_ = 7.19 and 10.19 µM).

Elmorsy et al. synthesized novel pyridopyrazolo-triazine and pyridopyrazolo-triazole derivatives and evaluated their cytotoxic activity against colon, hepatocellular, breast, and cervical carcinoma cell lines [155]. Compounds **155** and **156** demonstrated good cytotoxic activity against MCF7 and HepG2 (IC_50_ = 3.89 and 8.42 µM). Additionally, compound **157** showed the highest inhibitory activity against HCT116 and HeLa (IC_50_ = 7.71 and 13.11 µM).

Hossan et al. prepared and characterized several novel functionalized pyrazolopyrimidine compounds and evaluated their in vitro antiproliferation activity against HepG2, Hep2, MCF7, and PC3 cancer cell lines and normal WI38 cells [156]. Compounds **158** and **159** displayed remarkable inhibitory activities toward all tested cancer cells with IC_50_ values ranging from 18.31 to 39.98 µM compared to the reference drug 5-fluorouracil.

## 7. Future Perspective

Pyrazoles, as a class of nitrogen-containing heterocyclic compounds, possess abundant nitrogen atoms and aromatic rings in their core scaffolds, making them extremely promising lead structures for anticancer drug discovery. In recent years, various pyrazole derivatives have been extensively explored for anticancer drug development due to their unique chemical properties. Currently, pyrazole inhibitors targeting the epidermal growth factor receptor (EGFR) have attracted considerable attention. Future research on pyrazole derivatives as anticancer agents will likely focus on several key aspects: First, further optimization of the pyrazole core to improve binding affinity and selectivity against EGFR and other critical anticancer targets, such as HDAC, PI3K/AKT, and VEGFR; second, combination therapies with other anticancer agents to achieve synergistic therapeutic efficacy; third, development of antibody-drug conjugates utilizing the advantages of pyrazole small molecules to enhance tumor targeting; fourth, design of PROTACs and other novel targeted anticancer modalities; fifth, generation of new pyrazole compounds to overcome drug resistance mechanisms. In summary, pyrazole scaffolds hold tremendous potential for enabling impactful advances in anticancer drug discovery, and are poised to spearhead the development of a new generation of potent and safe anticancer therapeutics.

## 8. Conclusions

Over the past few decades, drug discovery has become integral to medical research. The remarkable chemical diversity of heterocyclic compounds has attracted substantial scientific attention worldwide. Among them, nitrogen-containing heterocyclic pyrazole derivatives possess a broad spectrum of biological activities and therapeutic promise as prospective anticancer agents due to their unique chemical structures. In recent years, numerous pyrazole derivatives have been synthesized to target tumor-associated proteins including kinases, microtubule proteins, topoisomerases, and COX-2. Their anticancer activities were evaluated accordingly. Certain pyrazole compounds exhibited potent selective inhibition against targets, such as BRAF, EGFR, and other kinases. Additionally, pyrazole derivatives with varied structural skeletons demonstrated antiproliferative and pro-apoptotic effects across multiple cancer cell lines. Currently, some pyrazoles have advanced to clinical trials for treating several malignant cancers. However, most pyrazole anticancer candidates remain at the preclinical stage, necessitating further optimization to enhance efficacy and reduce toxicity. The pyrazole scaffold continues to be an important source for anticancer drug discovery. Other novel derivatives will likely find clinical applications in cancer treatment in coming years. Here, we comprehensively review and analyze over 150 individual pyrazole derivatives, providing a useful resource to stimulate more research into anticancer drug discovery using this versatile scaffold.

## Figures and Tables

**Table 1:** Some commercially available drugs containing pyrazole skeleton.

Drug	Structure	Principal Indications	Mechanism of Action
Lonazolac [10]	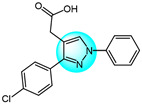	Rheumatoid arthritis; osteoarthritis	Lonazolac elicits its therapeutic effects primarily through preferential inhibition of COX-2, leading to reduced prostaglandin synthesis and blocking of inflammatory processes that mediate pain and fever.
Celecoxib [11]	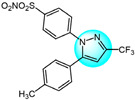	Rheumatoid arthritis; osteoarthritis	Celecoxib is a highly selective inhibitor of COX-2, which leads to reduced production of proinflammatory prostaglandins such as PGE2.
Crizotinib [12]	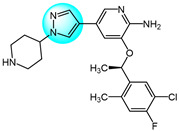	Advanced non-small cell lung cancer	Crizotinib is a highly selective inhibitor of ALK and ROS1 tyrosine kinases. It blocks downstream signaling pathways involved in cellular proliferation and anti-apoptosis.
Difenamizole [13]	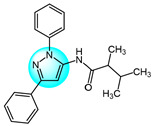	Acute and chronic arthritis; soft tissue inflammation	Difenamizole is a selective inhibitor of tyrosine phosphorylation of COX-2. It suppresses the phosphorylation of tyrosine residues on COX-2, thereby inhibiting its activity. This reduces levels of the inflammatory mediators endoperoxides and prostaglandins produced from the cyclooxygenase pathway.
Rimonabant [14]	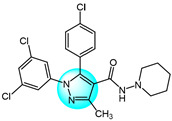	Overweight and obesity	Rimonabant is a selective antagonist of the cannabinoid CB1 receptor. It selectively blocks CB1 receptors in both the central nervous system and peripheral tissues.
Apixaban [15]	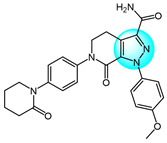	prevention of venous thromboembolism	Apixaban is a selective and reversible inhibitor of coagulation factor Xa. It inhibits factor Xa activity, thereby blocking thrombin generation and blood coagulation.
Fezolamine [16]	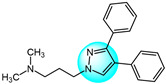	Hypertension; pain	Fezolamine is a non-selective α adrenergic receptor antagonist. It inhibits vasoconstriction mediated by the catecholamines adrenaline and noradrenaline.
Sidenafil [17]	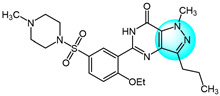	Erectile dysfunction; pulmonary arterial hypertension	Sildenafil selectively inhibits phosphodiesterase type 5 (PDE5), the enzyme responsible for breaking down cyclic guanosine monophosphate (cGMP).
Betazole [18]	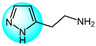	Diagnosis of impaired gastric acid secretion	Betazole is a highly selective H2 receptor agonist. It binds to and activates H2 receptors with high affinity, stimulating parietal cells to release gastric acid.
Fomepizole [19]	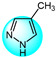	Severe ethanol intoxication	Fomepizole is a potent competitive inhibitor of alcohol dehydrogenase. By inhibiting this enzyme, fomepizole blocks the metabolism of ethanol, thereby reducing the formation of the toxic metabolite acetaldehyde.
Indiplon [20]	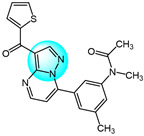	Sleep disorders	Indiplon acts as a positive allosteric modulator of GABAA receptors. It increases the receptor’s affinity for GABA, facilitating inhibitory synaptic transmission mediated by GABA.
Zaleplon [21]	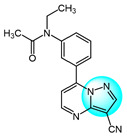	Circadian rhythm sleep disorders	Zaleplon selectively binds to the α1 subunit of the GABAA receptor. By increasing the receptor’s affinity for GABA, it facilitates inhibitory synaptic transmission mediated by GABA binding to the GABAA receptor.
Tepoxalin [22]	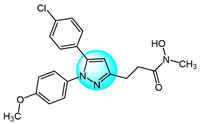	Rheumatoid arthritis	Tepoxalin is a dual inhibitor of cyclooxygenase (COX) and 5-lipoxygenase (5-LOX). It inhibits the production of pro-inflammatory mediators including prostaglandins and leukotrienes.
Deracoxib [23]	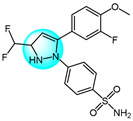	Pain and inflammation associated with osteoarthritis in dogs	Deracoxib is a highly selective inhibitor of cyclooxygenase-2 (COX-2). It inhibits the COX-2 mediated synthesis of prostaglandins.
Pyrazofurin [24]	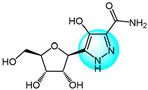	Acute and chronic myeloid leukemia	Pyrazofurin is an inhibitor of purine nucleotide synthesis. It inhibits the enzyme orotate phosphoribosyltransferase (OMPdecase) in tumor cells, blocking the synthesis of pyrimidine nucleotides and inhibiting DNA and RNA synthesis.
Surinabant [25]	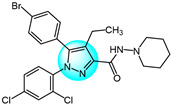	Was previously developed for treating tobacco dependence and obesity (currently has no approved therapeutic indications)	Surinabant is a selective inverse agonist of the cannabinoid CB1 receptor. It binds to CB1 receptors and inhibits their constitutive activity.

## Data Availability

No new data were created in this study. All the data reported in this review were found in original articles cited in the text.
